# A Single-Cell Transcriptomics CRISPR-Activation Screen Identifies Epigenetic Regulators of the Zygotic Genome Activation Program

**DOI:** 10.1016/j.cels.2020.06.004

**Published:** 2020-07-22

**Authors:** Celia Alda-Catalinas, Danila Bredikhin, Irene Hernando-Herraez, Fátima Santos, Oana Kubinyecz, Mélanie A. Eckersley-Maslin, Oliver Stegle, Wolf Reik

**Affiliations:** 1 Epigenetics Programme, Babraham Institute, Cambridge CB22 3AT, UK; 2 Wellcome Trust Sanger Institute, Hinxton, Cambridge CB10 1SA, UK; 3 European Molecular Biology Laboratory, Genome Biology Unit, Heidelberg 69117, Germany; 4 Division of Computational Genomics and Systems Genetics, German Cancer Research Center (DKFZ), Heidelberg 69120, Germany; 5 European Molecular Biology Laboratory, European Bioinformatics Institute, Hinxton, Cambridge CB10 1SA, UK; 6 Centre for Trophoblast Research University of Cambridge, Cambridge CB2 3EG, UK

**Keywords:** CRISPRa, scRNA-seq, single cell, screen, zygotic genome activation, ZGA, MOFA, *Dppa2*, *Smarca5*, *Patz1*

## Abstract

Zygotic genome activation (ZGA) is an essential transcriptional event in embryonic development that coincides with extensive epigenetic reprogramming. Complex manipulation techniques and maternal stores of proteins preclude large-scale functional screens for ZGA regulators within early embryos. Here, we combined pooled CRISPR activation (CRISPRa) with single-cell transcriptomics to identify regulators of ZGA-like transcription in mouse embryonic stem cells, which serve as a tractable, *in vitro* proxy of early mouse embryos. Using multi-omics factor analysis (MOFA+) applied to ∼200,000 single-cell transcriptomes comprising 230 CRISPRa perturbations, we characterized molecular signatures of ZGA and uncovered 24 factors that promote a ZGA-like response. Follow-up assays validated top screen hits, including the DNA-binding protein *Dppa2*, the chromatin remodeler *Smarca5*, and the transcription factor *Patz1*, and functional experiments revealed that *Smarca5’*s regulation of ZGA-like transcription is dependent on *Dppa2*. Together, our single-cell transcriptomic profiling of CRISPRa-perturbed cells provides both system-level and molecular insights into the mechanisms that orchestrate ZGA.

## Highlights

• Large-scale pooled CRISPR-activation screen with single-cell RNA-seq for 230 genes
• MOFA+ identified 24 screen hits that induced a ZGA-like signature
• Nine genes were independently validated as regulators of ZGA-like transcription
• *Smarca5* regulates ZGA-like transcription in a *Dppa2*-dependent manner

## Graphical Abstract

**Figure:**
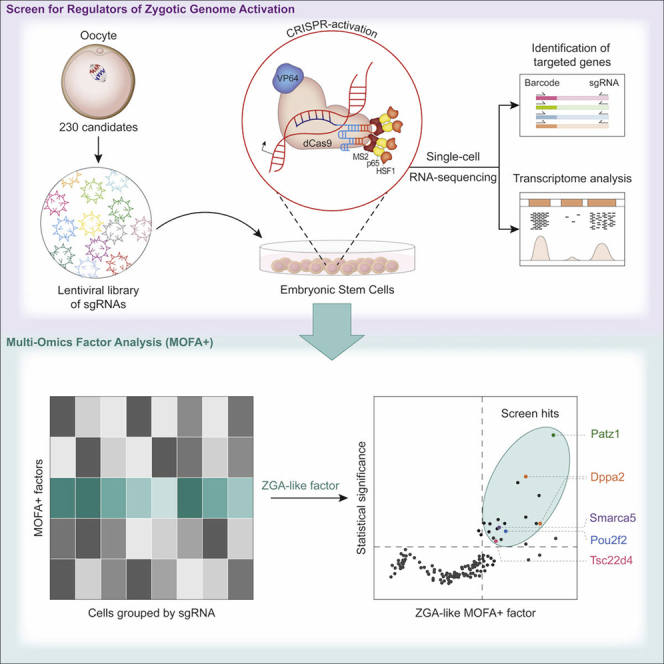

## Teaser

Alda-Catalinas et al. developed a pooled CRISPR-activation screen combined with single-cell transcriptomics to identify regulators of zygotic genome activation (ZGA) using an *in vitro* proxy of early mouse embryos, embryonic stem cells. After interrogation of 230 candidate regulators, 24 hits were identified using multi-omics factor analysis (MOFA+), including the transcription factor *Patz1*, the DNA-binding protein *Dppa2*, and the chromatin remodeler *Smarca5*. Follow-up functional experiments demonstrated that *Smarca5’*s regulation of ZGA-like transcription is dependent on *Dppa2*.

## Introduction

Zygotic genome activation (ZGA) is the first transcriptional event that takes place in an embryo (reviewed in Vastenhouw et al., 2019) and is a critical step in early development. In mouse, following an initial minor wave of ZGA in the late zygote, the major wave of ZGA occurs at the mid-to-late two-cell embryo stage and is characterized by the transcriptional activation of thousands of genes (reviewed in Vastenhouw et al., 2019; Jukam et al., 2017; Svoboda, 2018; Yartseva and Giraldez, 2015). In addition to the transcriptome, the epigenetic and chromatin landscapes are drastically remodeled during this transition, including reprogramming of histone post-translational modifications, global chromatin accessibility and three-dimensional (3D) structure, and global DNA demethylation (reviewed in Fraser and Lin, 2016; Eckersley-Maslin et al., 2018; Jansz and Torres-Padilla 2019). However, while several regulators of ZGA have previously been identified (reviewed in Eckersley-Maslin et al., 2018), a comprehensive understanding of the complex regulation of the transcriptional and epigenetic events that occur during ZGA remains elusive.

Recent studies have shown the power of combining pooled CRISPR-Cas9-based screening with single-cell RNA sequencing (scRNA-seq) to obtain a comprehensive readout of the perturbations introduced, enabling interrogation of gene function and regulation at a cellular level in an unbiased manner (Jaitin et al., 2016; Dixit et al., 2016; Adamson et al., 2016; Datlinger et al., 2017; Xie et al., 2017; Genga et al., 2019; Gasperini et al., 2019; Replogle et al., 2020). However, most of these studies have exclusively considered loss-of-function perturbations through CRISPR knockout (KO) (Jaitin et al., 2016; Dixit et al., 2016; Datlinger et al., 2017) or CRISPR-interference (CRISPRi) (Adamson et al., 2016; Xie et al., 2017; Genga et al., 2019; Gasperini et al., 2019). Consequently, these existing approaches can only be used to interrogate genes that are already expressed in the cellular system under study (Gilbert et al., 2014). CRISPR activation (CRISPRa) is a potent tool for selective transcriptional upregulation of endogenous genes, which functions by targeting a dead Cas9 (dCas9) with transcriptional co-activators to gene promoters using short-guide RNAs (sgRNAs) (Cheng et al., 2013; Gilbert et al., 2014; Chavez et al., 2015; Konermann et al., 2015) and has been successfully used for cellular reprogramming and the study of cellular transitions (Chakraborty et al., 2014; Black et al., 2016; Liu et al., 2018; Weltner et al., 2018; Yang et al., 2019; Genga et al., 2019). CRISPRa is preferable to traditional overexpression techniques, such as cloned cDNA overexpression, as it leads to target gene activation at physiologically relevant levels (Chavez et al., 2015; Sanson et al., 2018; Yang et al., 2019). Moreover, as it does not require cloning of genes, CRISPRa is highly scalable and allows the activation of genes that are, otherwise, difficult to clone or transfect into cells (Konermann et al., 2015; Horlbeck et al., 2016; Joung et al., 2017).

High-throughput screening in preimplantation mouse embryos is not feasible due to the scarcity of material, maternal stores of proteins and complex manipulation techniques required. Recent studies have shown that a ZGA-like state can be mimicked in mouse embryonic stem cells (ESCs) (Zalzman et al., 2010; Macfarlan et al., 2012; Bošković et al., 2014; Ishiuchi et al., 2015; Akiyama et al., 2015; Eckersley-Maslin et al., 2016; Rodriguez-Terrones et al., 2018). Consequently, these cells represent an ideal system for *in vitro* screening and have been previously used to identify regulators of ZGA (Rodriguez-Terrones et al., 2018; Fu et al., 2019; Yan et al., 2019; Eckersley-Maslin et al., 2019). While most of these studies probing ZGA regulators in ESCs have focused on repressors (Rodriguez-Terrones et al., 2018; Fu et al., 2019), positive inducers of ZGA have thus far not been interrogated in a high-throughput systematic manner. Such regulators are more relevant given the transcriptionally inactive state prior to ZGA and can be identified in ESCs by assessing the transcriptional changes triggered downstream of their overexpression (Eckersley-Maslin et al., 2019). Furthermore, these screening systems developed for the identification of ZGA-like regulators have relied on the use of a ZGA promoter-driven fluorescent protein as a reporter (Rodriguez-Terrones et al., 2018; Fu et al., 2019; Yan et al., 2019; Eckersley-Maslin et al., 2019) without a systematic analysis of ZGA genes.

Here, we developed a high-throughput CRISPRa screening method that combines pooled sgRNA delivery with a transcriptomic readout at single-cell resolution, enabling systematic identification of key inducers of transcriptional activation events. We applied this technology to probe candidate regulators of ZGA-like transcription in ESCs. Using integrative dimensionality reduction based on multi-omics factor analysis (MOFA+), thereby assessing both coding and non-coding transcriptomic changes, we identified maternal factors that induce ZGA-like transcription, including the transcription factor *Patz1*and the chromatin remodeler *Smarca5,* as well as previously known regulators, such as the DNA-binding protein *Dppa2* (De Iaco et al., 2019, Eckersley-Maslin et al., 2019, Yan et al., 2019). Furthermore, we mechanistically dissected a key part of the ZGA regulation network, revealing that *Smarca5* regulates ZGA-like transcription via *Dppa2*.

## Results

### A CRISPRa Screen for ZGA-like Regulators at Single-Cell Resolution

**Figure 1:**
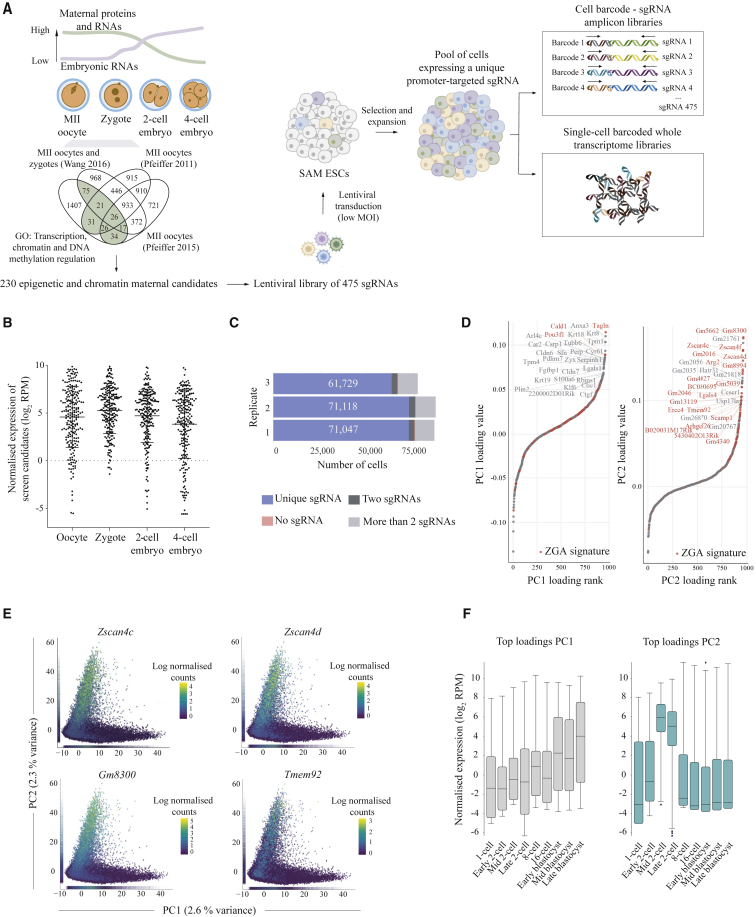
A CRISPRa Screen for ZGA-like Regulators at Single-Cell Resolution (A) Schematic overview of the single-cell CRISPRa screen, highlighting the selection of candidates, lentiviral transduction strategy, and generation of 10x Genomics 3′ scRNA-seq libraries and barcoded sgRNA amplicon libraries. (B) Dot-plot showing normalized expression levels (log_2_ reads per million; RPM) of the screening candidates in oocytes, zygotes, two-cell, and four-cell embryos. Data analyzed from Xue et al. (2013). (C) Number of cells expressing a unique sgRNA (blue), two sgRNAs (dark gray), more than two sgRNAs (light gray), or none (pink) in each of the three transduction replicates. The number of cells assigned to a unique sgRNA in each replicate is displayed. (D) Genes ranked by the loadings of PC1 (left) and PC2 (right), highlighting in red previously know ZGA genes (as described in Table S2; see also Table S3 for gene loading values). (E) PC analysis displaying a scatterplot of the first two PCs (PC1 versus PC2) with cells colored by the expression of the ZGA markers *Zscan4c*, *Zscan4d*, *Gm8300,* and *Tmem92*. Marginal distributions of PC1 and PC2 values are displayed as rug plots along the respective axis. (F) Box-whisker plots showing normalized expression levels (log_2_ reads per million; RPM) for the top 50 loadings for PC1 (gray) and PC2 (light blue) during preimplantation development (data analyzed from Deng et al., 2014) (see Table S3 for gene loadings). As expected in serum-grown ESCs, PC1 loadings peak at blastocyst stages whereas PC2 loadings peak at mid-to-late two-cell embryo stages, identifying this component as ZGA-like.

To systematically identify regulators of ZGA-like transcription, we developed a high-throughput pooled screening method that combines CRISPRa with single-cell transcriptomics (Figure 1A). Given the large-scale epigenetic and transcriptional changes that occur during the maternal-to-zygotic transition (reviewed in Eckersley-Maslin et al., 2018), we hypothesized that maternal epigenetic and transcriptional factors have crucial roles in regulating ZGA and, consequently, we focused our screen on such candidate regulators. Due to the technical limitations of high-throughput screening in early embryos, we used mouse ESC as an *in vitro* proxy to mimic ZGA (Eckersley-Maslin et al., 2018, 2019; Rodriguez-Terrones et al., 2018; Fu et al., 2019; Yan et al., 2019), reasoning that overexpression of ZGA regulators may induce a ZGA-like transcriptional signature which can be captured by scRNA-seq.

Our screening method builds on the robust and potent CRISPRa system “synergistic activator mediator” (SAM CRISPRa) (Konermann et al., 2015) in which in addition to dCas9-VP64, high activation levels are achieved by recruiting the trans-activators p65 and heat shock factor 1 (HSF1), both fused to a MS2 RNA-binding protein, through MS2 loops contained within the sgRNA scaffold sequence. We generated serum-grown clonal ESCs expressing both dCas9-VP64 and MS2-p65-HSF1 constitutively (referred to as SAM ESC), which have a largely unchanged transcriptome compared with parental E14 ESCs (Figure S1A), indicating that expression of the CRISPRa machinery does not substantially alter gene expression. To optimize and validate our screening system, we initially carried out a pilot experiment considering two strong positive regulators, the murine endogenous retrovirus with leucine tRNA primer (MERVL) (Yang et al., 2020) and the zinc finger and SCAN domain-containing 4 (*Zscan4*) gene cluster (Zhang et al., 2019), thereby confirming that CRISPRa can be used to induce a ZGA-like signature in mouse ESCs detectable by scRNA-seq. Briefly, SAM ESCs were transduced with sgRNAs targeting either MERVL long terminal repeats (LTRs) or *Zscan4* gene cluster promoters (Table S1), and single-cell transcriptomes were captured using the 10x Genomics scRNA-seq 3′ polyA-primed platform (Figures S1B–S1D, see STAR Methods). These key markers of ZGA are expressed in a low proportion of ESCs (Macfarlan et al., 2012; Zalzman et al., 2010; Eckersley-Maslin et al., 2016). CRISPRa significantly increased the proportion of cells expressing MERVL and *Zscan4* 3-fold (3.49% to 10.43%) and 2.8-fold (18.63% to 52.84%), respectively, compared with a non-targeting sgRNA control (Figures S1E and S1F). Interestingly, MERVL LTR activation led to *Zscan4* upregulation and vice versa (Figures S1E and S1F), suggesting synergistic regulation as part of a network. We then defined a ZGA signature based on 2,115 genes described in the literature to be expressed in the mouse embryo during ZGA or in the ZGA-like state of mouse ESCs (Eckersley-Maslin et al., 2016; Hendrickson et al., 2017; Li et al., 2018) (Table S2). We found that the proportion of cells expressing these ZGA transcripts increased 4.28-fold and 3.34-fold upon MERVL LTR and *Zscan4* CRISPRa, respectively (∼2% of cells transduced with a non-targeting sgRNA control to 8.56% of cells transduced with MERVL LTR sgRNAs and 6.67% of cells transduced with *Zscan4* sgRNAs) (Figure S1G). The upregulation of ZGA genes upon MERVL LTR activation is consistent with MERVL LTRs acting as functional promoters driving the expression of hundreds of chimaeric ZGA transcripts (Macfarlan et al., 2012; Huang et al., 2017; Franke et al., 2017; Yang et al., 2020). Similarly, *Zscan4c* cDNA overexpression has recently been shown to induce the expression of ZGA genes (Eckersley-Maslin et al., 2019; Zhang et al., 2019). Collectively, the results from this pilot experiment not only validated 10x Genomics scRNA-seq as a suitable readout of ZGA-like expression following CRISPRa of relevant regulators but also enabled us to estimate that approximately 400 cells per sgRNA (power 0.8, corrected p value < 0.0005, see STAR Methods) are required to detect a ZGA-like transcriptional response upon CRISPRa of a positive hit exerting a similar effect to MERVL and *Zscan4* activation.

Next, we applied our screening method to an extensive set of candidate regulators of ZGA shortlisted using publicly available proteomic datasets (Pfeiffer et al., 2011, 2015; Wang et al., 2016) and gene ontology enrichment (see STAR Methods). In total, we considered 230 proteins present in MII oocytes and zygotes with roles in transcription and epigenetic regulation (Figure 1A; Table S1), which are expressed prior to and at the time of ZGA (Figure 1B). Next, we designed a pooled sgRNA library containing two sgRNAs for each of the 230 candidate maternal ZGA regulators, targeting the 180-bp window upstream of the transcription start site (TSS), along with fifteen non-targeting sgRNA controls (Konermann et al., 2015; Joung et al., 2017) (Table S1). The resulting library consisting of 475 sgRNAs was cloned into a lentiviral vector modified from the CRISPR droplet sequencing (CROP-seq) method (Datlinger et al., 2017) to include MS2 loops in the sgRNA scaffold sequence (referred to as CROP-sgRNA-MS2, Figure S1H; see STAR Methods). This lentiviral vector backbone enables both CRISPRa via SAM and capture of the sgRNA target sequence in 10x Genomics 3′ scRNA-seq libraries. Notably, the 475 sgRNAs were represented in the cloned plasmid library (Figure S1I; Table S1).

SAM ESCs were transduced with this lentiviral library of 475 sgRNAs at a <0.1 multiplicity of infection (MOI) in triplicate (Figure S2A). Following selection and expansion of the pool of transduced cells, single-cell transcriptomes were generated using the 10x Genomics scRNA-seq 3′ polyA-primed platform, and their corresponding sgRNAs were further amplified using a specific amplification protocol (Hill et al., 2018) (Figure 1A; see STAR Methods). A total of 341,103 single-cell transcriptomes were sequenced across three transduction replicates (see STAR Methods). After scRNA-seq quality controls (Figures S2B–S2D), sgRNA assignment to each individual cell and removal of cells with no or multiple sgRNAs assigned, we obtained a total of 203,894 cells expressing a unique sgRNA for downstream analysis (Figure 1C; see STAR Methods). All sgRNAs were captured consistently across the three replicates, with an average coverage of 437 cells per sgRNA for the combined dataset (Figures S2E and S2F). The number of cells expressing each sgRNA (Table S1) matched the representation distribution of the sgRNA plasmid library (Figure S2G), indicating that activation of the target genes did not have any strong effects on cell proliferation or viability.

For an initial exploration of the sources of variation in our dataset, we applied principal component analysis (PCA). While gene ontology enrichment of the top 50 gene loadings of the first principal component (PC1) identified this component as capturing intrinsic variation in cell-to-cell contacts and cell shape (Figure 1D; Table S3), excitingly, the second component (PC2) robustly captured variation of genes that are highly expressed in mid-to-late two-cell embryos at the time of ZGA, including *Zscan4c*, *Zscan4d*, *Gm8300*, and *Tmem92* (Figures 1D–1F; Tables S2 and S3). This was consistent between replicates (Figure S2H), validating the robustness of our screen. Together, these results indicate that our CRISPRa scRNA-seq screen, with the selected maternal candidates, induced expression variation that mimics a ZGA-like transcriptional response in ESCs, suggesting that a substantial fraction of our candidates did indeed induce a ZGA-like gene signature.

### Identification of Activators of a ZGA-like Transcriptional Signature Using MOFA+

**Figure 2:**
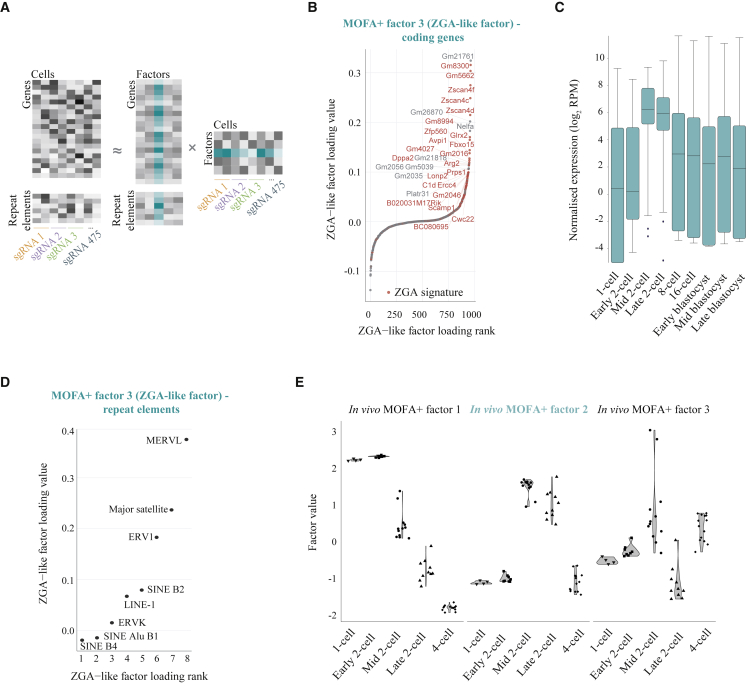
Identification of a ZGA-like Transcriptional Signature Using MOFA+ (A) Schematic of the joint analysis of coding gene and repeat element expression using multi-omics factor analysis (MOFA+). Data matrices of dimension features (genes or repeat elements) in cells grouped by sgRNA expression are treated as distinct views in the model and decomposed into the product of weights (or loadings) and factors. Factor 3 in the trained model, interpreted as a ZGA-like factor, is highlighted in green. (B) Coding genes ranked by their loadings of MOFA+ factor 3, highlighting in red previously known ZGA genes (as described in Table S2; see also Table S4 for gene loading values), indicating that this factor captures a ZGA-like response, and thus, identifying it as a MOFA+ ZGA-like factor. (C) Box-whisker plots showing normalized expression levels (log_2_ reads per million; RPM) for the top 50 gene loadings of MOFA+ factor 3 (ZGA-like factor) during preimplantation development (data analyzed from Deng et al. (2014) (see Table S4 for gene loadings). (D) Repeat element families ranked by their loadings of MOFA+ factor 3 (ZGA-like factor). (E) Violin plots for MOFA+ factor values 1–3 trained on scRNA-seq data for zygotes, early two-cell, mid two-cell, late two-cell, and four-cell stage embryos (data analyzed from Deng et al., 2014).

Next, we set out to characterize the observed ZGA-like transcriptional signature in more detail. In addition to coding genes, we also included transposable or repeat elements in our analysis (Figures S3A and S3B; see STAR Methods), since they are key drivers of gene expression during early embryonic development, and more specifically, ZGA (reviewed in Rodriguez-Terrones and Torres-Padilla, 2018). We used MOFA+ (Argelaguet et al., 2018, 2020) to combine the expression of coding genes and repeat elements within a single model and to disentangle individual activating sgRNAs responsible for inducing the observed ZGA-like response (see STAR Methods; Box 1). Briefly, the MOFA+ framework allows integration of both data modalities, coding genes and transposable or repeat elements, as distinct views, while also accounting for different groups of cells as manifested by expression of different sgRNAs (Box 1). The model identifies the most important (unobserved) factors that explain the transcriptional variability within and between sets of cells with specific sgRNA expression (Figure 2A; see STAR Methods; Box 1). Excitingly, among the MOFA+ factors identified (Table S4), factor 3 again captured a ZGA-like transcriptional signature: the coding genes with the highest loadings for factor 3 are enriched in ZGA genes and highly expressed in mid-to-late two-cell embryos (Figures 2B, 2C, and S3C–S3F) and, among the repeat classes analyzed, the ZGA-related MERVL repeat (Macfarlan et al., 2012) was most prominently associated to factor 3 (Figures 2D and S3G). Other MOFA+ factors captured technical and biological variability associated with ESC cultures rather than specific transcriptional programs in preimplantation development (Figure S3E), including protein metabolism and cell-cycle events captured by factor 1 or epigenetic heterogeneity captured by factor 2 (Rulands et al., 2018) (Table S4).

Box 1 **Using MOFA+ to Identify Transcriptional Signatures in scRNA-Seq Pooled CRISPR Screens**

MOFA, which was first developed in 2018 (Argelaguet et al., 2018), is a computational method that discovers the principal sources of variation in multi-omics data. One of the key features of this method is the ability to capture a complex high-dimensional dataset by a small number of latent sources of variation—named “factors”—which are jointly reconstructed across multiple data modalities, or “views.” For instance, MOFA has been used to understand the relationship between three molecular layers or “views” (transcription, DNA methylation, and chromatin accessibility) during germ-layer commitment in mouse embryos (Argelaguet et al., 2019).

Within its improved version, MOFA+ (Argelaguet et al., 2020), multiple advances have been brought together, making the method applicable to single-cell omics data. One of them is the ability to account for structures across single cells by combining cells into “groups.”

Considered together, MOFA+ features make it a great fit for the analysis of pooled CRISPR screens with complex readouts, such as the CRISPRa scRNA-seq screen described here. In this type of screens, cells have the distinctive feature of expressing a certain sgRNA targeting a screen candidate, and this sgRNA can be read in the scRNA-seq libraries. Cells are first grouped by the sgRNA assigned to them (these are the “groups” in the model). This allows each “group” of cells expressing the same sgRNA to be modeled with separate hyperparameters, enabling the discovery of latent “factors” that can later be linked to one or multiple sgRNAs.

Furthermore, taking advantage of the multi-modal nature of MOFA+, we quantified the expression of repeat or transposable element families and used this quantification as a second “view” in the model, allowing us to integrate protein-coding and non-coding transcription in the “factors” discovered. This is particularly important when considering ZGA-like transcriptional signatures, which are also driven by repeat elements, such as MERVL. By doing this, we made sure that we identified hits that not only regulate the expression of ZGA-like protein-coding genes but also ZGA-like repeat elements. In the model, each gene or each repeat element family that contribute to “view” 1 and “view” 2, respectively, is a “feature.”

In our analysis, factor 3 could be clearly identified to capture variability related to ZGA because its top “loadings” (that is, the “features” that showed the highest contribution to the variance explained by this factor) were enriched in genes and repeat elements known to be expressed at the time of ZGA. “Factor” 3 or ZGA-like “factor” was subsequently used to call screen hits. For each sgRNA, we fitted a regression model between the sgRNA targeting activity (cells expressing a given targeting sgRNA versus cells expressing non-targeting sgRNA controls) and the value of MOFA+ “factor” 3. This approach enabled us to infer the effect size for each targeting sgRNA, that is, the extent to which each targeting sgRNA and, consequently, the activation of the targeted screen candidate, upregulated a ZGA-like transcriptional signature.

**Figure:**
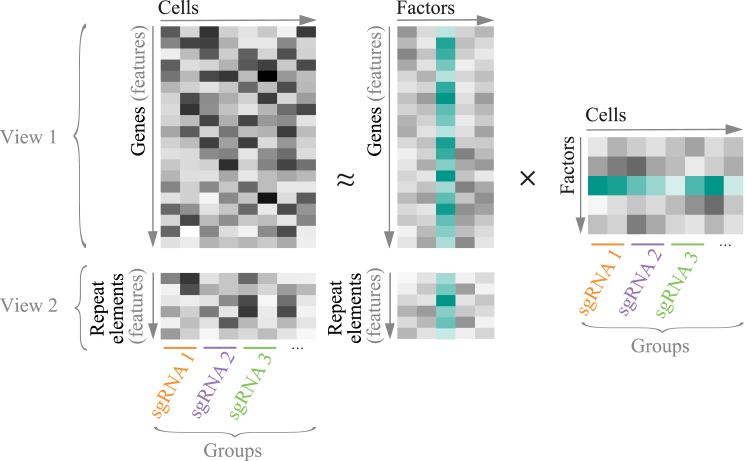

We further assessed MOFA+ as a method to identify ZGA-like signatures by applying the same approach to an *in vivo* mouse preimplantation scRNA-seq dataset (Deng et al., 2014). We included samples for zygotes, early, mid, and late two-cell embryos and four-cell embryos in the model, therefore, providing ample temporal resolution to disentangle transcriptional events around and at the time of ZGA (see STAR Methods, Table S5). While MOFA+ factor 1 ordered the scRNA-seq samples by developmental stage, the second MOFA+ factor clearly distinguished pre- (zygotes and early two-cell embryos) and post-ZGA stages (four-cell embryos) from ZGA stages (mid and late two-cell embryos) (Figures 2E and S3H) and, similarly to the MOFA+ ZGA-like factor (factor 3) from our CRISPRa perturbation dataset on ESCs, it captured previously described ZGA genes (Table S2) among the top gene loadings (Figure S3I). Together, these analyses support the robustness of our screening strategy, demonstrate that there are maternal factors among our selected candidates that induce ZGA-like transcription and validate MOFA+ as a statistical approach for the unbiased identification of relevant gene signatures.

**Figure 3:**
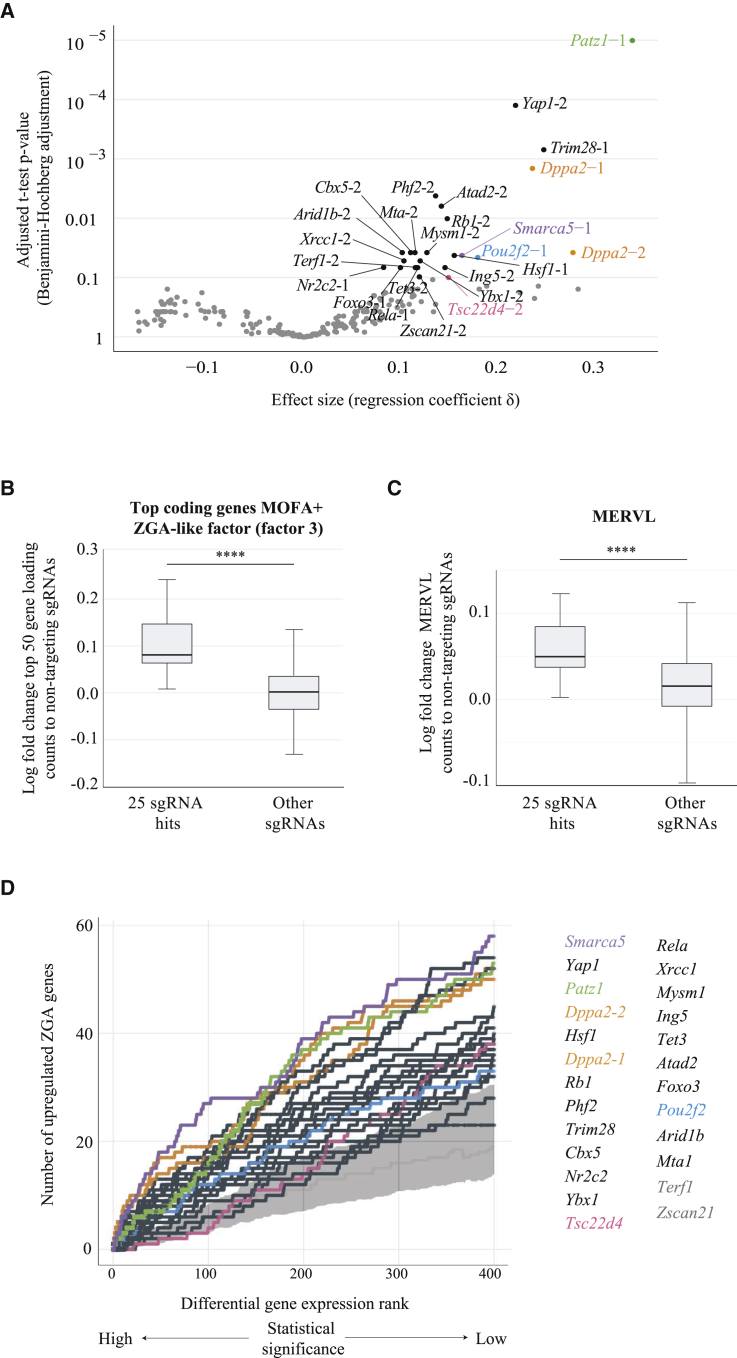
Identification of Activators of a ZGA-like Transcriptional Signature (A) Screen hit rank shown as the effect size (regression coefficient value δ) and the adjusted t test p value (Benjamini-Hochberg adjustment). Target genes with sgRNA(s) at FDR <10% (25 sgRNAs) were considered hits, and their names are displayed (see Table S1 for the full ranking), with *Patz1* (green), *Dppa2* (orange), *Smarca5* (purple), *Pou2f2* (blue), and *Tsc22d4* (pink) sgRNA hits highlighted. (B) Box-whisker plots showing log fold change expression for the top 50 genes associated with MOFA+ factor 3 (ZGA-like factor, ranked absolute loadings) in cells expressing the 25 sgRNA hits and cells expressing other targeting sgRNAs, compared to cells expressing non-targeting sgRNA controls. Expression is quantified in normalized counts (^∗∗∗∗^p value = 3.7 × 10^−10^, Mann-Whitney two-tailed test). (C) Box-whisker plots showing log fold change of MERVL normalized counts in cells expressing the 25 sgRNA hits and cells expressing other targeting sgRNAs, compared to cells expressing non-targeting sgRNA controls (^∗∗∗∗^p value = 8.2 × 10^−7^, Mann-Whitney two-tailed test). (D) Cumulative rank of the number of ZGA signature genes (as described in Table S2) upregulated by each sgRNA hit compared to non-targeting sgRNA controls, considering the top 400 genes ranked by statistical significance of differential gene expression test (generalized linear model likelihood ratio test as implemented in EdgeR). In gray is shown an empirical background distribution estimated based on differential gene expression between cells with non-targeting sgRNA controls, displaying plus and minus one standard deviation around the mean of ZGA signature genes recovered by non-targeting sgRNAs. The names of the target genes for sgRNAs identified as hits in A) are depicted, with those for which the differential gene expression rank overlaps with the non-targeting control background shown in gray. *Patz1* (green), *Dppa2* (orange), *Smarca5* (purple), *Pou2f2* (blue), and *Tsc22d4* (pink) sgRNA hits are highlighted.

To reveal individual candidate genes that induced a ZGA-like signature when activated, we assessed the extent to which the MOFA+ ZGA-like factor (factor 3) is associated with the expression of individual targeting sgRNAs. Specifically, for each sgRNA, we fitted a regression model between the sgRNA targeting activity (cells expressing a given targeting sgRNA versus cells expressing non-targeting sgRNA controls) and the activity profile of MOFA+ factor 3 (see STAR Methods), which enabled us to infer the effect size (or regression coefficient δ) for each targeting sgRNA (Figure 3A; Table S1). For this analysis, we considered 228 sgRNAs with any evidence for gene activation of the corresponding gene (out of 460 targeting sgRNAs in the pooled library or 49.6%, mean log_2_ fold change of target gene to non-targeting sgRNA control > 0) (Table S1). Excitingly, this identified 25 sgRNAs for which CRISPRa of the corresponding gene induced ZGA-like transcription (false discovery rate [FDR] < 10%) (Figure 3A; Table S1). These 25 sgRNA hits targeted 24 unique genes, with both sgRNAs targeting *Dppa2* identified as hits.

Consistent with the detected association with MOFA+ ZGA-like factor (factor 3), cells expressing the 25 sgRNA hits specifically induced the expression of genes linked to this factor (Figures 3B and S4A), while genes associated to other MOFA+ factors remained largely unaltered (Figure S4B), highlighting the specificity of these screen hits in upregulating a ZGA-like signature. Similarly, the ZGA-associated MERVL elements (Macfarlan et al., 2012), major satellite repeats (Casanova et al., 2013) and ERV1 (Zhang et al., 2019), but not other repeat families, were upregulated by these sgRNA hits (Figures 3C and S4C), consistent with these repeat families ranking top in the MOFA+ ZGA-like factor (Figure 2D).

Lastly, we investigated individual target genes induced by CRISPRa of the 25 sgRNA hits compared to non-targeting sgRNA controls (see STAR Methods). When assessing differential gene expression between targeted and non-targeted cells transcriptome-wide, only a small subset of genes was significantly differentially expressed for most hits (between 1 and 160, median 7; FDR < 0.1, Table S1; see STAR Methods). However, ranking of the top 400 upregulated genes by statistical significance identified the downstream genes of 23 out of the 25 sgRNA hits (92%) as being prominently enriched for known ZGA transcripts (compared to background enrichment estimated from differential gene expression between cells with non-targeting sgRNA controls) (Figure 3D; Tables S1 and S2). This result obtained using a complementary analysis strategy provides additional confidence into our method for hit calling based on MOFA+, highlights its advantages in capturing relevant gene signatures, of both coding and non-coding transcription, in an unbiased way, and shows that MOFA+ can identify screen hits that would otherwise be missed using conventional differential gene expression analyses, due to lack of power to detect the effects on individual genes. In summary, using MOFA+ to integrate the expression of coding genes and transposable elements in our CRISPRa scRNA-seq dataset, we identified 24 genes whose activation induced a ZGA-like transcriptional response.

Among these, we identified three previously known maternal ZGA regulators, namely the transcription factors *Yap1* (Yu et al., 2016) and *Hsf1* (Christians et al., 2000) and the DNA-binding protein *Dppa2* (Eckersley-Maslin et al., 2019; De Iaco et al., 2019; Yan et al., 2019). Excitingly, we also identified 21 maternal proteins that have not been previously linked to ZGA. Among these, there are transcription factors, such as *Patz1*, *Pou2f2*, *Foxo3*, or *Tsc22d4*; histone lysine demethylases, such as *Phf2*; histone methylation readers, such as *Ing5*; DNA demethylases, such as *Tet3*; heterochromatin proteins, such as *Cbx5* (also known as HP1α); chromatin remodelers from the SWItch/Sucrose Non-Fermentable (SWI/SNF) complex, such as *Arid1b*, the Imitation SWItch (ISWI) complex, such as *Smarca5*, or the nucleosome remodeling and deacetylase (NuRD) complex, such as *Mta1*; DNA-repair proteins, such as *Xrcc1*; or nuclear receptors, such as *Nr2c2*. Interestingly, network analysis of these 24 hits revealed known interactions between several of these ZGA-like regulators (Figure S4D), suggesting that ZGA regulation is a process coordinated between multiple transcriptional and epigenetic factors.

### Validation of Screen Hits by Arrayed CRISPRa

**Figure 4:**
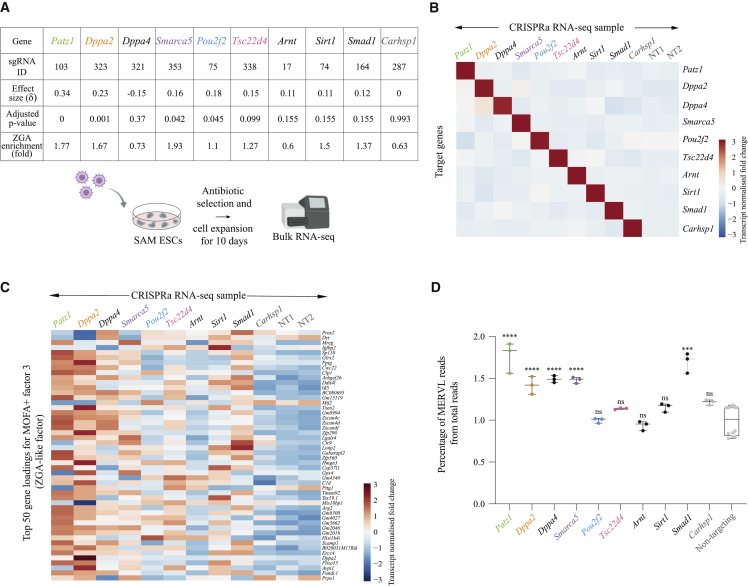
Validation of Screen Hits by Arrayed CRISPRa (A) Top: MOFA+ parameters (effect size and adjusted p value) and ZGA gene enrichment (based on analysis described in Figure 3D) for the screen hits *Patz1*, *Dppa2*, *Smarca5*, *Pou2f2*, and *Tsc22d4*, the candidates *Dppa4*, *Arnt*, *Sirt1*, and *Smad1*, and the negative control candidate *Carhsp1*. Bottom: schematic representation of an arrayed CRISPRa validation approach followed by bulk polyA-capture RNA-seq to confirm the screen hits *Patz1*, *Dppa2*, *Smarca5*, *Pou2f2*, and *Tsc22d4* and to interrogate the candidates *Dppa4*, *Arnt*, *Sirt1*, and *Smad1*, using *Carhsp1* as a negative control. (B) Heatmap showing normalized gene expression, scaled per gene, of the target genes interrogated by arrayed CRISPRa and bulk RNA-seq. Controls are two different non-targeting sgRNAs (NT1 and NT2). (C) Heatmap showing normalized gene expression, scaled per gene, of the top 50 gene loadings for MOFA+ factor 3 (ZGA-like factor) in bulk RNA-seq libraries for *Patz1*, *Dppa2*, *Dppa4*, *Smarca5*, *Pou2f2*, *Tsc22d4*, *Arnt*, *Sirt1*, *Smad1*, and *Carhsp1* CRISPRa. Controls are two different non-targeting sgRNAs (NT1 and NT2). (D) Box-whisker plots showing expression of the MERVL repeat family in percentage of total reads measured by bulk RNA-seq after CRISPRa of *Patz1* (green), *Dppa2* (orange), *Dppa4* (black), *Smarca5* (purple), *Pou2f2* (blue), *Tsc22d4* (pink), *Arnt* (black), *Sirt1* (black), *Smad1* (black), and *Carhsp1* (gray) and in two non-targeting sgRNA controls (gray). Each dot represents a biological replicate. Statistically significant differences to controls are reported as ^∗∗∗∗^p value < 0.0001, ^∗∗∗^p value < 0.001, ns (non-significant): p value > 0.05; Mann-Whitney two-tailed test.

Next, we considered 10 selected candidate ZGA regulators for further validation using arrayed CRISPRa with sgRNAs used in the screen, followed by transcriptomic analysis assessed by bulk polyA-capture RNA-seq, in biological triplicates (Figure 4A; Table S1). In addition to five screen hits identified by MOFA+ (*Patz1*, *Dppa2*, *Smarca5*, *Pou2f2*, and *Tsc22d4*; FDR < 10%) (Figure 4A), we also included three candidate genes (*Arnt*, *Sirt1*, and *Smad1*) that ranked highly when considering MOFA+ effect size (δ) or the analysis based on ZGA gene enrichment (Figure 3D; Table S1) but failed to meet the statistical significance criterion in our primary analysis (FDR < 10%) (Figure 4A; Table S1). Interestingly, this was also the case for previously known ZGA regulators, such as *Gata3* (effect size δ = 0.24, adjusted p value = 0.14, 1.27-fold ZGA gene enrichment) (Figure S4A; Table S1) (Eckersley-Maslin et al., 2019). *Dppa4* has been shown to be a potent regulator of ZGA-like transcription together with its partner *Dppa2* (Eckersley-Maslin et al., 2019; De Iaco et al., 2019; Yan et al., 2019); however, both of its targeting sgRNAs, while inducing target gene activation, did not pass the statistical threshold to be considered screen hits nor they ranked highly by effect size or ZGA gene enrichment (Figures 4A, S5A, and S5B; Table S1). Nevertheless, *Dppa4* was included in this validation dataset (Figure 4A) to assess the discrepancy between our screen data and published literature. Lastly, we also included *Carhsp1* as a negative control since it ranked low in the screen rank while both of its targeting sgRNAs showed effective target gene activation (Figures 4A, S5A, and S5B; Table S1).

For each of the 10 genes tested, CRISPR-induced transcriptional activation was specific to the targeting sgRNA (Figure 4B). Downstream of target gene activation and consistent with the screen scRNA-seq data and our hit calling strategy, the hits *Patz1*, *Dppa2*, *Smarca5*, *Pou2f2*, and *Tsc22d4* induced upregulation of the ZGA genes captured in MOFA+ factor 3 (Figure 4C). Similarly, *Dppa4*, *Arnt*, *Sirt1*, and *Smad1*, but not the negative control *Carhsp1*, induced ZGA-like transcription compared with non-targeting sgRNA controls (Figure 4C). Furthermore, the repeat family of MERVL elements, which captured the highest variability among the repeat families in the scRNA-seq dataset as analyzed by MOFA+ (Figure 2D), was significantly upregulated by the screen hits *Patz1*, *Dppa2*, and *Smarca5* and the candidates *Dppa4* and *Smad1*, but not by *Pou2f2*, *Tsc22d4*, *Arnt*, *Sirt1*, or the negative control *Carhsp1* (Figure 4D). This confirms our hit calling strategy and suggests that we have identified a set of 24 highly confident ZGA-like regulators. The fact that some candidates that were below our cutoff for statistical significance (FDR < 10%) induced a clear ZGA signature, such as *Dppa4*, *Arnt*, *Sirt1*, or *Smad1*, also shows that methods such as bulk RNA-seq may still have advantages compared with scRNA-seq-based screens, in particular for identifying weaker regulators.

### *Patz1*, *Dppa2*, and *Smarca5* Are Potent Inducers of ZGA-like Transcription

Both the screen scRNA-seq data as well as the validation experiments revealed that the hits *Patz1*, *Dppa2*, and *Smarca5* strongly upregulate ZGA-like transcripts. For these targets, we found that, despite the increased power in calling differential gene expression using bulk RNA-seq, the transcriptional changes captured by scRNA-seq and bulk RNA-seq upon CRISPRa showed consistent patterns (Figure S5C), demonstrating that CRISPRa coupled with scRNA-seq readout is a robust method to assess the transcriptional responses triggered by gene overexpression.

**Figure 5:**
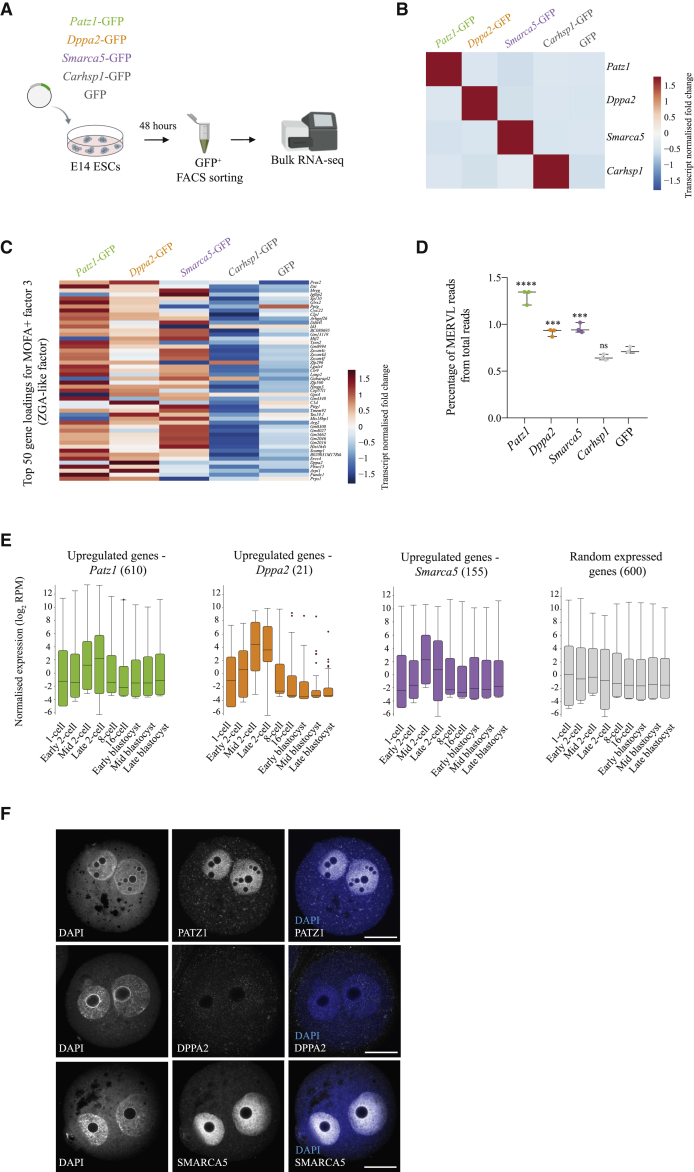
*Patz1*, *Dppa2*, and *Smarca5* Are Potent Inducers of ZGA-like Transcription (A) Schematic representation of a complementary validation approach for *Patz1*, *Dppa2*, and *Smarca5*, using *Carhsp1* as a negative control, consisting of cDNA-eGFP transient transfections into mouse ESCs followed by eGFP^+^ fluorescence-activated cell sorting (FACS) and bulk polyA-capture RNA-seq. (B) Heatmap showing normalized gene expression, scaled per gene, of *Patz1*, *Dppa2*, *Smarca5*, and *Carhsp1* in bulk RNA-seq libraries after cDNA overexpression of these genes, compared with an eGFP-only transfection. (C) Heatmap showing normalized gene expression, scaled per gene, of the top 50 gene loadings for MOFA+ factor 3 (ZGA-like factor) in bulk RNA-seq libraries for *Patz1*, *Dppa2*, *Smarca5,* and *Carhsp1* cDNA overexpression. The control is a eGFP-only transfection. (D) Box-whisker plots showing expression of the MERVL repeat family in percentage of total reads measured by bulk RNA-seq after cDNA overexpression of *Patz1* (green), *Dppa2* (orange), *Smarca5* (purple), and *Carhsp1* (gray). The control is an eGFP-only transfection (gray). Each dot represents a biological replicate. Statistically significant differences to eGFP-only control are reported as ^∗∗∗∗^p value < 0.0001, ^∗∗∗^p value < 0.001, ns (non-significant): p value > 0.05; Mann-Whitney two-tailed test. (E) Box-whisker plots showing normalized expression levels (log_2_ reads per million; RPM) of differentially upregulated genes by both arrayed CRISPRa and cDNA overexpression of *Patz1* (green), *Dppa2* (orange), and *Smarca5* (purple) as well as a random set of expressed genes (gray) during preimplantation development (data analyzed from Deng et al., 2014). Differential gene expression was calculated with EdgeR (FDR < 0.05). The number of analyzed genes in each case is depicted in brackets. (F) Representative single optical slices of zygotes immunostained for PATZ1, DPPA2, and SMARCA5, showing single channels and composites with DAPI. Scale bars represent 25 μm.

To further validate the role of these factors as ZGA-like regulators, we used an alternative method of gene overexpression by transfecting cDNA-eGFP fusion constructs into ESCs and investigated the transcriptional response by bulk polyA-capture RNA-seq in biological triplicates (Figure 5A). We also included *Carhsp1*-eGFP cDNA transfection as a negative control. Upon verifying successful gene overexpression (Figure 5B), we first compared, genome-wide, the transcriptional response induced by CRISPRa of these genes to that induced by cDNA overexpression, and we observed a markedly similar pattern across methods (Figure S5D). Additionally, the three screen hits, *Patz1*, *Dppa2*, and *Smarca5*, triggered similar genome-wide transcriptional changes, which were clearly distinct from the changes induced by *Carhsp1* (Figure S5D), suggesting they regulate similar transcriptional networks.

Next, we analyzed ZGA-like transcription and revealed that cDNA overexpression of *Patz1*, *Dppa2,* and *Smarca5*, but not *Carhsp1*, led to upregulation of ZGA genes, including those captured by the MOFA+ ZGA-like factor (Figure 5C). Moreover, and consistent with the CRISPRa scRNA-seq and bulk RNA-seq data (Figures 3C and 4D), MERVL was significantly upregulated by *Patz1*, *Dppa2*, and *Smarca5*, but not *Carhsp1*, cDNA overexpression (Figure 5D). Interestingly, LINE-1 expression, which has also been linked to ZGA (Percharde et al., 2018), was induced by *Patz1* and *Dppa2* (Figure S5E).

Genes significantly upregulated by both CRISPRa and cDNA overexpression of *Patz1*, *Dppa2,* and *Smarca5* (see STAR Methods) were highly expressed at the time of ZGA *in vivo* (Figure 5E), providing strong evidence that these maternal regulators activate a ZGA-like program in ESCs. Furthermore, and in agreement with our initial candidate selection based on proteomic datasets (Pfeiffer et al., 2011, 2015; Wang et al., 2016), we confirmed protein expression of these factors in zygotes by immunofluorescence, showing strong pronuclear localization of SMARCA5 and both pronuclear and cytoplasmic expression of PATZ1 and DPPA2 (Figure 5F). In summary, we confirmed three top screen hits as regulators of ZGA-like transcription, which also validates our method as a reliable high-throughput tool for detecting positive regulators of transcriptional programs.

### *Smarca5* Requires *Dppa2* to Induce ZGA-like Transcription

Our results reveal numerous key regulators of ZGA-like transcription, including transcription factors and chromatin remodelers. Therefore, we sought to understand the interdependencies between them focusing on two of the strongest inducers, *Dppa2* and *Smarca5*, aiming to obtain a deeper understanding of the interplay between transcriptional activation and chromatin remodeling during ZGA. *Dppa2* has recently been identified as a potent inducer of ZGA networks (Eckersley-Maslin et al., 2019; De Iaco et al., 2019; Yan et al., 2019). *Smarca5*, which we identified as one of the top screen hits and independently validated as a ZGA-like regulator, is the ATP-ase subunit of ISWI chromatin remodeling complex.

**Figure 6:**
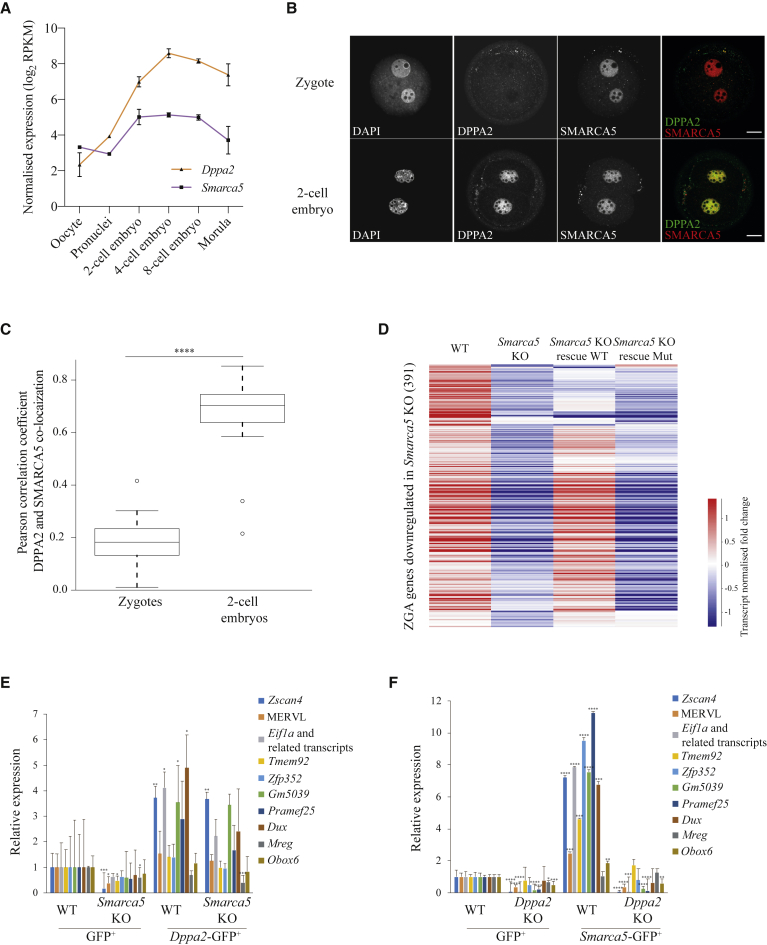
*Smarca5* Requires *Dppa2* to Induce ZGA-like Transcription (A) Normalized expression levels (log_2_ reads per kilobase per million; RPKM) of *Dppa2* (orange, triangles) and *Smarca5* (purple, squares) in oocytes and preimplantation development (data analyzed from Xue et al., 2013). Data are shown as mean plus standard deviation of biological replicates. (B) Representative single optical slices of zygotes (top row) and two-cell stage embryos (bottom row) immunostained for DPPA2 and SMARCA5, showing single channels and composites. Scale bars represent 20 μm. (C) Box-plots showing Pearson correlation coefficients calculated for co-localization of DPPA2 and SMARCA5 in the pronuclei of 10 zygotes and in the nuclei of 10 two-cell stage embryos. Co-localization values in the two pronuclei in zygotes and nuclei of each blastomere in two-cell embryos were measured separately. DPPA2 and SMARCA5 co-localize in two-cell embryos but not in zygotes (^∗∗∗∗^p value < 0.0001, Mann-Whitney two-tailed test). (D) Heatmap showing normalized expression, scaled per gene, of downregulated ZGA genes in *Smarca5* KO mouse ESCs compared to WT (EdgeR, FDR < 0.05), in WT ESCs, *Smarca5* KO ESCs, and *Smarca5* KO ESCs expressing a *Smarca5* WT protein or a *Smarca5* catalytically dead mutant protein (Mut) (data analyzed from Barisic et al., 2019). (E and F) Analysis of relative expression levels of ZGA-like transcripts by quantitative reverse transcription PCR in (E) WT and *Smarca5* KO mouse ESCs after 48-h transient transfection of eGFP or *Dppa2*-eGFP and (F) WT and *Dppa2* KO mouse ESCs after 48-h transient transfection of eGFP or *Smarca5*-eGFP. eGFP^+^ cells were FACS-sorted before gene expression analysis. Relative expression levels are normalized to WT cells transfected with eGFP and sorted for eGFP^+^. Data are shown as mean plus standard deviation of three biological replicates. Statistically significant differences to WT GFP^+^ control are reported (^∗∗^p value < 0.01, ^∗∗∗^p value < 0.001, ^∗∗∗∗^p value < 0.0001; absence of stars (non-significant): p value > 0.05; homoscedastic two-tailed t test).

*Smarca5* knockdown in zygotes reduces transcription of a set of genes (Torres-Padilla and Zernicka-Goetz, 2006), but its role as a ZGA regulator has not yet been characterized. Both *Dppa2* and *Smarca5* are expressed at the mRNA level throughout preimplantation development, with *Smarca5* being more highly expressed in the oocyte than *Dppa2*, which increases in its expression from the two-cell stage (Figure 6A). At the protein level, in zygotes, while DPPA2 localizes mostly in the cytoplasm, SMARCA5 is present in both pronuclei (Figures 5F and 6B). However, in two-cell embryos, at the time of ZGA, DPPA2 translocates to the nucleus and both proteins co-localize (Figures 6B and 6C). Notably, DPPA2 and SMARCA5 proteins have been shown to physically interact in ESCs (Hernandez et al., 2018). These observations suggest DPPA2 and SMARCA5 may function together to regulate their ZGA target genes. Consistent with our overexpression results, analysis of recently published *Smarca5* KO transcriptomic data (Barisic et al., 2019) revealed that loss of *Smarca5* led to downregulation of ZGA transcripts (Figure 6D). Similarly, recent studies have also shown that *Dppa2* KO ESCs lack expression of ZGA-like genes (Eckersley-Maslin et al., 2019; De Iaco et al., 2019; Yan et al., 2019). We next investigated whether *Smarca5* exerts its ZGA regulatory function through its catalytic ATPase activity or through interactions with accessory subunits of the ISWI complex. Analysis of published RNA-seq data of *Smarca5* KO ESCs (Barisic et al., 2019) revealed that wild type (WT), but not a catalytically dead *Smarca5* mutant, was able to restore expression of the 391 ZGA genes downregulated upon *Smarca5* loss (Figure 6D). This result indicates that the regulation of ZGA by *Smarca5* is dependent on its ATPase activity.

Finally, given their co-localization in two-cell embryos (Figures 6B and 6C), physical interaction in ESCs (Hernandez et al., 2018), and similar transcriptional effects (Figure S5D), we sought to understand the interdependencies, if any, between *Dppa2* and *Smarca5* through a series of KO and overexpression experiments analyzed by quantitative reverse transcription PCR. First, to test whether *Smarca5* is required for *Dppa2*’s function, we overexpressed *Dppa2*-eGFP in WT and *Smarca5* KO cells (Figures S6A and S6B). As shown by RNA-seq (Figure 6D), expression of ZGA genes was downregulated in *Smarca5* KO cells, although not completely lost (Figure 6E). Excitingly, expression of these genes was partially rescued by *Dppa2* overexpression (Figure 6E), while the pluripotency gene *Oct4* remained unaltered (Figure S6C). This suggests that *Dppa2* may act downstream or independently to *Smarca5* to regulate ZGA-like transcription. To test between these options, we overexpressed *Smarca5*-eGFP in WT and *Dppa2* KO ESCs (Figures S6D and S6E). As expected, in WT cells, *Smarca5* overexpression strongly induced the expression of ZGA genes, including the *Zscan4* cluster and MERVL (Figures 6F and S6F), whereas the pluripotency gene *Oct4* remained unchanged (Figure S6G). Interestingly, in *Dppa2* KO ESCs, which have nearly absent levels of ZGA transcripts (Eckersley-Maslin et al., 2019) (Figure 6F), ZGA-like gene expression could not be induced by *Smarca5*-eGFP overexpression (Figure 6F). This suggests *Dppa2* is required for *Smarca5*-mediated regulation of ZGA. Therefore, while *Smarca5* requires *Dppa2* for its ZGA-like transcriptional effects, *Dppa2* does not require *Smarca5*. Notably, *Smarca5* induced *Dppa2* expression but not vice versa (Figures 4B, S6B, and S6E). In conclusion, these experiments suggest that *Dppa2* and *Smarca5* may act together to induce a ZGA-like signature in mouse ESCs via chromatin remodeling and activation of transcription.

## Discussion

Here, we developed a pooled CRISPRa screen coupled with scRNA-seq readout and applied this high-throughput tool to identify regulators of ZGA. Our dataset, comprised of 203,894 single ESCs each expressing a unique sgRNA, allowed us to investigate the transcriptional consequences following CRISPRa of 230 maternally expressed epigenetic and transcriptional factors (Figures 1A–1C). By performing integrative dimensionality reduction of the expression of both coding mRNAs and repeat elements using MOFA+ (Figure 2A), we revealed 24 maternal factors that induced a transcriptional response reminiscent of the major wave of ZGA (Figures 3A–3D). Nine candidate regulators were experimentally validated as inductors of ZGA-like transcription in ESCs by arrayed CRISPRa (Figures 4C and 4D), among which *Patz1*, *Dppa2*, and *Smarca5* were further validated using complementary experimental approaches (Figures 5C–5F). These data not only validate our screen approach but also the roles of these proteins in regulating ZGA-like expression. Finally, we disentangled the interdependencies between *Dppa2* and *Smarca5* suggesting that *Smarca5* requires *Dppa2* to regulate ZGA (Figures 6E and 6F).

Our screening method provides a robust way to systematically interrogate a large number of genes for their effects on specific transcriptional programs, and we anticipate that it will be widely adaptable to many other biological contexts and research questions. While overexpression screens using traditional open reading frame (ORF) libraries have previously been coupled with an scRNA-seq readout (Parekh et al., 2018), CRISPRa has key advantages over traditional cDNA overexpression. First, target genes are upregulated at physiologically relevant levels (Chavez et al., 2015; Sanson et al., 2018; Yang et al., 2019). Second, it allows activation of genes that might be otherwise difficult to clone as well as activation of other genomic features, such as repeat elements (Figure S1E). Despite the differences in gene dosage and experimental design (Figures 4A and 5A), in our validation experiments with arrayed CRISPRa and cDNA overexpression, the transcriptional changes triggered by our top hits *Patz1*, *Dppa2*, and *Smarca5* are remarkably similar in the induction of ZGA-like gene expression (Figures 4C, 5C, and S5D), confirming CRISPRa as a robust method to analyze ZGA-like transcription.

Screening using single-cell transcriptomics has substantial advantages in terms of scalability and the possibility to disentangle cell-to-cell heterogeneity. However, it comes at a cost in terms of sensitivity for detecting transcriptional changes in individual cells (reviewed in Kelsey et al., 2017). Related work using CRISPR KO CROP-seq (Datlinger et al., 2017) applied computational downsampling analysis to show that as few as 12–13 cells per sgRNA can suffice to detect the expected transcriptional signatures upon deletion of T cell receptor signaling regulators. In our CRISPRa screen for ZGA-like regulators, we performed *a priori* power calculations and estimated that at least 400 cells were required per sgRNA to confidently detect a ZGA-like transcriptional response (Figure S1G; STAR Methods). While these design choices are highly dependent on the biological context under investigation, it is crucial to determine these parameters using prior knowledge or pilot data, as done in our study. Furthermore, to mitigate the reduced sensitivity of scRNA-seq, we considered a ZGA-like transcriptional signature rather than quantification of individual genes. To this end, our analysis builds on the MOFA+ framework (Argelaguet et al., 2020) to incorporate both the coding transcriptome and repetitive elements to define a robust and sensitive signature of ZGA-like transcriptional responses following CRISPRa (Figure 2A). We applied this model to 228 sgRNAs with evidence for gene activation of the corresponding target gene (Figure 3A; Table S1). Lack of detection of target gene activation for the remaining sgRNAs could be due to sub-optimal sgRNA design or technical dropouts that are commonly observed in scRNA-seq data. We demonstrate that our MOFA+ approach is superior in identifying relevant screen hits, compared with conventional differential gene expression analysis (Figures 3A and 3D; Table S1). Specific repeat classes are expressed during ZGA in mouse embryos, including MERVL (Kigami, 2003; Peaston et al., 2004; Macfarlan et al., 2012) and LINE-1 retrotransposons (Jachowicz et al., 2017; Percharde et al., 2018). Consistently, our top hits *Patz1*, *Dppa2*, and *Smarca5* all showed consistent upregulation of MERVL in the scRNA-seq data and validation experiments, and *Dppa2* and *Patz1* also upregulated LINE-1 expression (Figures 3C, 4D, 5D, and S5E), similar to what previous studies overexpressing ZGA-like regulators in ESCs have shown (Hendrickson et al., 2017; Eckersley-Maslin et al., 2019; De Iaco et al., 2017, 2019).

Of the 230 maternal candidates screened, 24 were identified as confident inducers of a ZGA-like response in ESCs (Figures 3A–3D). Among these hits, we identified factors that had been previously described as ZGA regulators, namely *Yap1* (Yu et al., 2016), *Dppa2* (Eckersley-Maslin et al., 2019; De Iaco et al., 2019; Yan et al., 2019), and *Hsf1* (Christians et al., 2000), further validating our screen approach. Several of our screen hits, such as *Phf2*, *Atad2*, *Cbx5*, *Smarca5*, *Terf1*, and *Tet3* have also been shown to significantly reduce the two-cell-like subpopulation that expresses ZGA-like transcripts in mouse ESCs after siRNA knockdown (Rodriguez-Terrones et al., 2018), consistent with an activator role of ZGA-like transcription for these factors. Consistent with our findings that *Smarca5* induces ZGA-like transcription, SMARCA5 localizes to sites of active transcription in zygotes, and its zygotic RNAi-mediated knockdown led to some defects in ZGA (Torres-Padilla and Zernicka-Goetz, 2006). Moreover, *Smarca5* homozygous KO embryos derived from heterozygous crosses arrest during preimplantation development (Stopka and Skoultchi, 2003). Excitingly, the majority of our screen hits, including the top-ranking *Patz1*, which we independently validated, have not been previously linked to ZGA, thus demonstrating the power of our approach. This included *Arid1b* (Figures 3A and 3D), a member of the SWI/SNF chromatin remodeling complex. In fact, *Smarca5* KO led to a reduction, but not complete elimination, of ZGA-like gene expression in ESCs (Figure 5E), suggesting there may be functional redundancy among chromatin remodelers in the early embryo at this crucial time of development.

While our primary analysis based on MOFA+ identified 24 highly confident screen hits, it is worth highlighting that candidates below the chosen significance threshold could still be validated as positive hits by bulk RNA-seq (Figure 4C), suggesting that an increased transcript detection sensitivity could provide an even larger hit list. This was the case for the candidates *Sirt1* and *Smad1,* and the previously described ZGA-like regulator *Dppa4* (Figure 4C) (Eckersley-Maslin et al., 2019; De Iaco et al., 2019; Yan et al., 2019). *Arnt*, which we also validated, had been implicated in but not specifically shown to regulate ZGA transcription (Guo et al., 2017).

In summary, we conclude that our CRISPRa single-cell transcriptomic screen has unraveled positive regulators of ZGA-like transcription. Our data and the hits identified open up many exciting new avenues for *in vivo* experiments testing the functional requirements, interdependencies, and redundancies between these regulators of ZGA-like expression. Furthermore, our CRISPRa followed by scRNA-seq screening method can be broadly applied in other biological contexts to systematically understand transcriptional regulation at the cellular level and identify positive regulators of key transcriptional programs in a large-scale and high-throughput manner.

## STAR★Methods

### Key Resources Table

**Table:** 

REAGENT or RESOURCE	SOURCE	IDENTIFIER
**Antibodies**		

Rabbit polyclonal anti-PATZ1	Abcam	Cat# ab154025
Rabbit polyclonal anti-SNF2H (SMARCA5)	Abcam	Cat# ab72499; RRID: AB_1270821
Mouse monoclonal anti-DPPA2, clone 6C1.2	Merck Millipore	Cat# mab4356; RRID: AB_1977389
Donkey anti-rabbit IgG, Alexa Fluor 568	Invitrogen	Cat# A10042; RRID: AB_2534017
Donkey anti-mouse IgG, Alexa Fluor 488	Invitrogen	Cat# A32766; RRID: AB_2762823

**Bacterial and Virus Strains**		

One Shot™ Stbl3™ Chemically Competent E. coli	Invitrogen	Cat# C737303
Library Efficiency™ DH5α Competent Cells	Invitrogen	Cat# 18263012
Individual sgRNA lentivirus cloned into lenti sgRNA(MS2)_puro backbone and into lenti CROP-sgRNA-MS2 backbone	This paper	N/A
Lentiviral sgRNA library cloned into CROP-sgRNA-MS2 backbone	This paper	N/A

**Biological Samples**		

N/A	N/A	N/A

**Chemicals, Peptides, and Recombinant Proteins**		

Murine LIF	Wellcome – MRC Cambridge Stem Cell Institute	https://www.stemcells.cam.ac.uk/research/facilities/tissueculture
TransIT transfection reagent	Mirus Bio	Cat# MIR2700
Polybrene	Millpore	Cat# TR-1003-G
LentiX Concentrator	Takara	Cat# 631231

**Critical Commercial Assays**		

Chromium Single Cell 3’ Library & Gel Bead Kit v2	10X Genomics	Cat# PN-120237
Chromium Single Cell A Chip Kit	10X Genomics	Cat# PN-120236
Chromium i7 Multiplex Kit	10X Genomics	Cat# PN-120262

**Deposited Data**		

Raw and quantified sequencing data: bulk RNA-seq data of E14 and SAM mouse ESCs	This paper	GEO: GSE135509 (https://www.ncbi.nlm.nih.gov/geo/query/acc.cgi?acc=GSE135509 )
Raw and quantified sequencing data: 10X Genomics 3’ scRNA-seq of MERVL LTR and *Zscan4* CRISPRa	This paper	GEO: GSE135554 (https://www.ncbi.nlm.nih.gov/geo/query/acc.cgi?acc=GSE135554)
Raw and quantified sequencing data: 10X Genomics CRISPRa screen dataset	This paper	GEO: GSE135621 (https://www.ncbi.nlm.nih.gov/geo/query/acc.cgi?acc=GSE135621)
Raw and quantified sequencing data: bulk RNA-seq of arrayed CRISPRa validations and bulk RNA-seq of *Patz1*, *Dppa2*, *Smarca5* and *Carhsp1* cDNA overexpression	This paper	GEO: GSE135512 (https://www.ncbi.nlm.nih.gov/geo/query/acc.cgi?acc=GSE135512)
Raw sequencing data: RNA-seq of mouse oocyte and preimplantation development	Xue et al., 2013	GEO: GSE44183
Raw sequencing data: RNA-seq of mouse preimplantation development	Deng et al., 2014	GEO: GSE45719
Raw sequencing data: RNA-seq of *Snf2h* (or *Smarca5*) KO mouse embryonic stem cells	Barisic et al., 2019	GEO: GSE112134
Mouse reference genome NCBI build 38, GRCm38 (mm10)	Genome Reference Consortium	https://www.ncbi.nlm.nih.gov/assembly/GCF_000001635.20/
Mouse repeat element annotation, GRCm38 (mm10)	RepeatMasker	http://www.repeatmasker.org

**Experimental Models: Cell Lines**		

Mouse: E14 embryonic stem cells	Hooper et al., 1987	RRID: CVCL_C320 https://discovery.lifemapsc.com/stem-cell-differentiation/in-vitro-cells/inner-cell-mass-mus-musculus-e14-university-of-edinburgh
Mouse: SAM embryonic stem cells	This paper	N/A
Mouse: *Dppa2* KO embryonic stem cells	Eckersley-Maslin et al., 2019	N/A
Mouse: *Smarca5* (or *Snf2h*) KO embryonic stem cells	Barisic et al., 2019	N/A
Human: HEK293T	ATCC	ATCC CRL-3216

**Experimental Models: Organisms/Strains**		

Mouse: C57Bl/6	N/A	N/A

**Oligonucleotides**		

Genomic PCR primers for dCas9-VP64 and MS2-p65-HSF1	This paper	Table S6
sgRNA protospacer sequences	Joung et al., 2017 and this paper	Table S1
Primers for amplicon sgRNA PCRs	Hill et al., 2018 and this paper	Table S6
Primers for PCR amplification of *Patz1*, *Dppa2*, *Smarca5* and *Carhsp1* cDNA sequences	This paper	Table S6
qRT-PCR primers	This paper	Table S6

**Recombinant DNA**		

pMD2.G	Didier Trono	Addgene plasmid #12259
psPAX2	Didier Trono	Addgene plasmid #12260
Lenti dCas9-VP64_Blast	Konermann et al., 2015	Addgene plasmid #61425
Lenti MS2-p65-HSF1_Hygro	Konermann et al., 2015	Addgene plasmid #61426
Lenti sgRNA(MS2)_puro	Konermann et al., 2015	Addgene plasmid #73795
Lenti CROP-sgRNA-MS2	This paper	Addgene plasmid #153457
pDONR221	Thermo Fisher Scientific	Cat# 12536017
pIG400_*Patz1* (cDNA)	This paper	N/A
pIG400_*Dppa2* (cDNA)	This paper	N/A
pIG400_*Smarca5* (cDNA)	This paper	N/A
pIG400_*Carhsp1* (cDNA)	This paper	N/A

**Software and Algorithms**		

CellRanger v2.1	Zheng et al., 2017	Github: https://github.com/10XGenomics/cellranger
Scanpy	Wolf et al., 2018	Github: https://github.com/theislab/scanpy
SAMtools	Li et al., 2009	http://www.htslib.org
BWA	Li and Durbin, 2009	http://bio-bwa.sourceforge.net
R	N/A	https://www.r-project.org/
MOFA+	Argelaguet et al., 2020	Github: http://bio-bwa.sourceforge.net
Trim Galore	Babraham Bioinformatics	www.bioinformatics.babraham.ac.uk/projects/trim_galore/
Hisat2	Kim et al., 2019	Github: https://github.com/DaehwanKimLab/hisat2
Bowtie2	Langmead and Salzberg, 2012	Github: https://github.com/BenLangmead/bowtie2
SeqMonk	Babraham Bioinformatics	https://www.bioinformatics.babraham.ac.uk/projects/seqmonk/
Volocity	Quorum Technologies	https://quorumtechnologies.com/volocity/volocity-downloads/downloads

**Other**		

Code for processing CRISPRa scRNA-seq screen dataset, including repeat element quantification and assignment of sgRNAs to cells	This paper	Github: https://github.com/gtca/crispra_zga

### Resource Availability

#### Lead Contact

Further information and requests for resources and reagents should be directed to and will be fulfilled by the Lead Contact, Wolf Reik (wolf.reik@babraham.ac.uk).

#### Materials Availability

CROP-sgRNA-MS2 plasmid has been deposited to Addgene (CROP-sgRNA-MS2, 153457).

#### Data and Code Availability

Sequencing data has been deposited in NCBI's Gene Expression Omnibus (Edgar et al., 2002) and are accessible through GEO Series accession number (GSE135622; https://www.ncbi.nlm.nih.gov/geo/query/acc.cgi?acc=GSE135622 ) under four sub-series:

- GSE135509 (https://www.ncbi.nlm.nih.gov/geo/query/acc.cgi?acc=GSE135509): Bulk RNA-seq data of E14 and SAM mouse ESCs.
- GSE135554 (https://www.ncbi.nlm.nih.gov/geo/query/acc.cgi?acc=GSE135554): 10X Genomics 3’ scRNA-seq of MERVL LTR and*Zscan4* CRISPRa.
- GSE135621 (https://www.ncbi.nlm.nih.gov/geo/query/acc.cgi?acc=GSE135621): 10X Genomics CRISPRa screen dataset.
- GSE135512 (https://www.ncbi.nlm.nih.gov/geo/query/acc.cgi?acc=GSE135512): Bulk RNA-seq of arrayed CRISPRa validations and bulk RNA-seq of*Patz1*, *Dppa2*, *Smarca5* and *Carhsp1* cDNA overexpression.

The code generated during this study is available in Github: https://github.com/gtca/crispra_zga

### Experimental Model and Subject Details

#### Cell Lines

All mouse embryonic stem cells (ESCs) were grown under serum/LIF conditions: DMEM (Gibco, 11995-040), 15% fetal bovine serum, 1 U/ml penicillin - 1 mg/ml streptomycin (Gibco, 15140-122), 0.1 mM nonessential amino acids (Gibco, 11140-050), 4 mM GlutaMAX (Gibco, 35050-061), 50 μM β-mercaptoethanol (Gibco, 31350-010), and 10^3^ U/ml LIF (Stem Cell Institute, Cambridge), and cultured at 37 °C in 5% CO_2_ on gelatinized tissue-culture plates. The media was refreshed every day and the cells passaged every other day with Trypsin EDTA (Thermo Fisher Scientific, 25200056).

SAM mouse embryonic stem cells (ESCs) were generated by lentiviral transduction of lenti dCas9-VP64_Blast (Addgene 61425) and lenti MS2-p65-HSF1_Hygro (Addgene 61426) into E14 mouse ESCs (male) (Hooper et al., 1987), followed by antibiotic selection and manual subcloning. Clones were checked by genomic PCR (primer sequences available in Table S6) and one clone was used for consecutive experiments.

*Dppa2* knock-out (KO) mouse ESCs were previously generated and described in Eckersley-Maslin et al., 2019 and *Smarca5* (also known as *Snf2h*) KO mouse ESCs in Barisic et al., 2019.

HEK293T cells (female) were grown in D10 media: DMEM (Gibco, 11995-040), 10% fetal bovine serum, 1 U/ml penicillin - 1 mg/ml streptomycin (Gibco, 15140-122) and cultured at 37 °C in 5% CO_2_ on T175 tissue culture flasks or 100mm tissue culture plates. The media was refreshed every other day and the cells passaged every three days with Trypsin EDTA (Thermo Fisher Scientific, 25200056).

#### Mice

All mice used in this study were C57Bl/6 and were bred and maintained in the Babraham Institute Biological Support Unit. All procedures were covered by a project license (to WR) under the Animal Scientific Procedures Act 1986, and are locally regulated by the Babraham Institute Animal Welfare, Experimentation, and Ethics Committee. Embryos were collected from C57Bl/6 females after superovulation and mating to C57Bl/6 males. Zygotes were collected on the day of plugging and two-cell embryos one day after plugging. The sex of embryos was not recorded at the time of collection because of their early developmental stage.

### Method Details

#### Candidate Selection for Primary Screen

Mouse proteins associated with nucleic acid binding and transcription factor activities were extracted from the PANTHER (http://pantherdb.org) (Mi et al., 2019) protein classes PC00171 and PC00218. The resulting gene list was intersected with proteins detected in mouse oocytes and zygotes in three different proteomic studies: 3,699 proteins in MII oocytes identified by Pfeiffer et al., 2011; 2,010 proteins identified simultaneously in MII oocytes of four inbred strains (129/Sv, C57Bl/6J, C3H/HeN, DBA/2J) by Pfeiffer et al., 2015; and 2,897 proteins detected in both MII oocytes and zygotes by Wang et al., 2016. Intersection of the four gene lists resulted in a list of 230 screen candidates (Figure 1A; Table S1).

#### Cloning

All sgRNAs used in this study were previously designed (Joung et al., 2017) to target the 180bp region upstream of the target gene TSS. Sequences are provided in Table S1.

The CROP-sgRNA-MS2 lentiviral backbone was synthesized by VectorBuilder by adapting the CROP-seq vector (Datlinger et al., 2017) with the following modifications: 1) the sgRNA scaffold sequence contains two MS2 loops that allow recruitment of MS2-p65-HSF1 in SAM ESCs; and 2) a fluorescent mCherry marker was included downstream of the EF1α promoter and linked through T2A to a puromycin resistance cassette, allowing assessment of the multiplicity-of-infection (MOI) by FACS and antibiotic selection of the cells (Figure S1H). This lentiviral backbone been deposited to Addgene (CROP-sgRNA-MS2, 153457).

For individual sgRNA cloning in pilot and validation experiments, two oligos were synthesized per sgRNA (Sigma Aldrich), one containing the protospacer sequence (Table S1) with a “CACCG” flank at the 5’ end and the other one synthesized as the reverse complementary sequence to the target sequence and flanked by “AAAC” at the 5’ end and by a “C” at the 3’ end. Each oligo pair was annealed using T4 Polynucleotide Kinase (PNK) enzyme (NEB, M0201S) and then cloned into the sgRNA(MS2)_puro backbone (Addgene 73795) or into the in-house built CROP-sgRNA-MS2 backbone (Figure S1H; Addgene 153457) by a Golden Gate reaction using BsmBI enzyme (Thermo Fisher Scientific, ER0451) and T7 ligase (NEB, M0318S). The product from the Golden Gate reaction was transformed into Stbl3 competent cells (Invitrogen, C737303). Between 2 to 3 colonies were picked per sgRNA and verified by Sanger sequencing.

For cloning the 475 sgRNA library into the CROP-sgRNA-MS2 backbone (Addgene 153457), first, oligos containing the sgRNA target sequence (Table S1), a 5’ end 26 base-pair (bp) flanking region complementary to the U6 promoter (TATCTTGTGGAAAGGACGAAACACCG) and a 3’ end 35 bp flanking region complementary to the sgRNA scaffold sequence (GTTTAAGAGCTAGGCCAACATGAGGATCACCCATG) were synthesized by Twist Bioscience. This oligo library was then amplified and cloned using Gibson assembly, as previously described (Joung et al., 2017), by VectorBuilder. Library coverage was estimated to be >11,000 folds by colony count of diluted transformations. 150 bp paired-end sequencing was performed on Illumina HiSeq4000 to analyse sgRNA representation in the library (Figure S1I).

cDNA-eGFP constructs were cloned by gateway cloning as previously described (Eckersley-Maslin et al., 2019). Sequence-verified cDNA sequences lacking stop codons were PCR-amplified from plasmids purchased from Thermo Fisher Scientific using forward primers containing an AttB1 sequence (GGGGACAAGTTTGTACAAAAAAGCAGGCTTCACC) followed by the 22 5’end bases of the cDNA of interest, and reverse primers containing an AttB2 sequence (GGGGACCACTTTGTACAAGAAAGCTGGGTC) followed by the 24 reverse complementary bases to the 3’ end of the cDNA sequence (Table S6). The PCR was performed with Phusion High-Fidelity DNA Polymerase (NEB, M0530S) or LongAmp Tap DNA Polymerase (NEB, M0323), following manufacturer’s instructions. The PCR product was then cloned into pDONR221 vector (Thermo Fisher Scientific, 12536017) using a Gateway BP clonase II enzyme (Thermo Fisher Scientific, 11789020) and DH5α competent cells (Invitrogen, 18263012), following manufacturer’s instructions. A Gateway LR II clonase reaction (Thermo Fisher Scientific, 11791100) was then used to transfer the cDNA sequences into an in-house built pDEST vector containing a CAG promoter and an in-frame C-terminal eGFP coding sequence linked to a blasticidin resistance marker by an IRES sequence, using DH5α competent cells (Invitrogen, 18263012). Expression plasmids were sequence verified by Sanger Sequencing prior to use.

#### Lentiviral Packaging and Titration

For lentiviral particle production, 3.5 million HEK293T cells were first seeded into 100mm tissue culture plates 24h before transfection. Next, they were co-transfected with 3.5 μg of pMD2.G (Addgene 12259), 6.5 μg of psPAX2 (Addgene 12260) and 10 μg of the lentiviral vector of interest: dCas9-VP64_Blast (Addgene 61425), MS2-p65-HSF1_Hygro (Addgene 61426), sgRNA(MS2)_puro (Addgene 73795) cloned with an individual sgRNA, or CROP-sgRNA-MS2 (Addgene 153457) cloned with an individual sgRNA. A single-tube reaction mix was prepared for each transfection, containing the three lentiviral plasmids and 60 μl of TransIT Reagent (Mirus Bio, 2700) diluted in 1.5 mL of Opti-MEM (Gibco, 31985), which was subsequently added drop-wise into the cells containing 8.5 mL of fresh media, following manufacturer’s instructions. 48h later, 10 mL of virus supernatant were harvested by filtering through a 0.45 μm filter (Sartorius, 16533) and supplemented with 8 μg/mL polybrene (Millpore, TR-1003-G). For sgRNA-expressing lentivirus from the CROP-sgRNA-MS2 backbone, the 10 mL of viral supernatant were concentrated 20x with LentiX Concentrator (Takara, 631231) due to the lower viral titer (Datlinger et al., 2017).

The lentiviral library of 475 sgRNAs was packaged by VectorBuilder: the plasmid library was co-transfected with a proprietary envelop plasmid encoding VSV-G and packaging plasmids encoding Gag/Pol and Rev into packaging cells. After a short incubation period, the supernatant was collected, followed by the removal of cell debris by centrifugation, filtration and PEG concentration of the viral particles.

To measure lentiviral titer, HEK293T cells were transduced with lentivirus diluted from the stock and then, a quantitative reverse transcription PCR-based approach was used to quantify the average number of integration events of the proviral genome per host genome.

#### Lentiviral Transductions

Individual sgRNAs were transduced into SAM ESCs by direct supplementation of the lentivirus into the medium for 24 hours. 48 hours after transduction, 1 μg/ml puromycin was added to the medium for selection of transduced cells. Cells were selected and passaged for 8 days after addition of puromycin before harvesting for bulk or 10X single-cell RNA-sequencing library preparation.

The lentiviral library of 475 sgRNAs cloned into the CROP-sgRNA-MS2 backbone (Addgene 153457) was transduced in triplicate into SAM ESCs by direct supplementation of the concentrated lentivirus into the medium for 24 hours. The transductions were done at <0.1 multiplicity-of-infection (MOI) (Figure S2A) into 5 million SAM ESCs, achieving a representation >1000 cells / sgRNA, considering that <10% of the cells were transduced. Two days after transduction, mCherry expression was analysed by flow-cytometry on BD LSR Fortessa and selection with 1 μg/ml puromycin started afterwards. Cells were selected and passaged for 8 days after addition of puromycin before harvesting for 10X single-cell RNA-sequencing library preparation.

#### Transient Transfections

cDNA-eGFP constructs were transfected into pre-plated E14, *Dppa2* KO, *Smarca5* KO or wild-type counterpart mouse ESCs in triplicates using Lipofectamine 2000 Reagent (Thermo Fisher Scientific, 11668019) and Opti-MEM (GIBCO, 31985), following manufacturer’s instructions. Cells were grown for 48 hours before eGFP^+^ FACS sorting on BD Influx High-Speed Cell Sorter or analysis on BD LSR Fortessa.

#### Preparation of scRNA-seq Libraries

A single cell suspension was loaded into the 10X Chromium device and libraries were prepared using the 10X Single Cell 3’ Library & Gel Bead Kit v2 (10X Genomics, PN-120237), following manufacturer’s instructions. In the pilot test, the following samples were loaded each in a lane of the 10X chip Chromium Controller: E14 ESCs, SAM ESCs transduced with the non-targeting sgRNA control 461, SAM ESCs transduced with the MERVL LTR sgRNAs 459 and 460 individually and pooled at the time of sequencing, and SAM ESCs transduced with the *Zscan4* sgRNAs 457 and 458 individually and pooled at the time of sequencing (see Table S1 for sgRNA sequences). Each 10X library was sequenced on an Illumina HiSeq4000 lane with 75 cycles for read 1, 75 cycles for read 2 and 8 cycles i7 sample index. 1,956, 2,045, 2,233, and 2,362 cells were captured, respectively, for each sample based on number of cell barcodes detected, with a total of 21,371 genes detected in the dataset, before quality control processing.

In the single-cell dataset of SAM ESCs transduced with the 475 sgRNA library, each transduction replicate was loaded across a full 10X chip Chromium Controller (8 lanes), with 20,000 cells per lane. Each 10X library was sequenced on an Illumina HiSeq4000 lane with 26 cycles for read 1, 98 cycles for read 2 and 8 cycles i7 sample index. A total of 114,866 cells were captured for replicate 1, 118,646 cells for replicate 2 and 107,591 cells for replicate 3 based on number of cell barcodes detected, which, after merging transduction replicates, resulted in a dataset of 341,103 cells with a total of 23,760 genes detected before quality control processing.

Amplicon sgRNA PCRs were performed for each of the 24 full length 10X cDNA samples of SAM ESCs transduced with the 475 sgRNA library, as previously described (Hill et al., 2018). Briefly, 10 ng of full length 10X cDNA were used as starting material and each round of PCR amplification with the primers described in Hill et al., 2018 (see also Table S6) was monitored by KAPA SYBR (KR0389) to avoid overcycling. After multiplexing, these enrichment sgRNA libraries were sequenced across two lanes of the Illumina Hiseq2500 Rapid Run, with 27 cycles for read 1, 267 cycles for read 2 and 8 cycles for i7 sample index.

#### Analysis of scRNA-seq Data from Pilot Test

##### Processing, Quality Control and Gene Expression Quantification

All 10X scRNA-seq data was processed with the default CellRanger v2.1 pipeline (Zheng et al., 2017) for mapping to the mm10 mouse genome assembly. Gene counts were further analysed with Scanpy (Wolf et al., 2018). For quality control of the samples from the pilot test after individual transductions with MERVL LTRs and *Zscan4* sgRNAs, cells with less than 15,000 UMI counts and/or less than 4,000 detected genes, cells with more than 40,000 UMI counts and/or more than 6,500 detected genes, and cells with more than 5% of UMI reads coming from mitochondrial genes were discarded (Figures S1B–S1D). After this quality control, 1,138 E14 ESCs, 687 SAM ESCs transduced with the non-targeting sgRNA control 461, 1,227 SAM ESCs transduced with the MERVL LTR sgRNAs 459 and 460 and 899 SAM ESCs transduced with the *Zscan4* sgRNAs 457 and 458 were retained for analysis. A gene was considered for downstream analysis if it was detected (UMI count > 0) in at least 10 cells that passed the quality control filter across the full dataset. The final dataset consisted of 16,498 genes across 3,951 cells. The number of UMIs for each cell and gene were adjusted by the library size in each cell, dividing by the total number of UMIs per cell. Gene expression levels were obtained as log-transformed adjusted UMI counts, scaled by a factor of 10,000. For quantification of MERVL repeat elements, see “repeat element quantification” section below.

##### Power estimation:number of cells to be sequenced per sgRNA

From the pilot experiment where SAM ESCs were transduced with sgRNAs targeting MERVL LTRs and *Zscan4* cluster promoters (Figures S1E and S1F), a power estimation was made to determine the number of single cells required to be sequenced per sgRNA in the CRISPRa screen to detect a ZGA-like signature in a positive hit. We used a qualitative two-tailed Fisher’s exact test considering that the percentage of cells that expressed ZGA-like transcripts was 2.04% in cells transduced with a non-targeting sgRNA control and 8.56% in cells transduced with a positive inductor (e.g. MERVL LTR sgRNAs) (Figure S1G). The test returned a sample size of 399 cells per sgRNA to detect a ZGA-like signature in a positive hit with an adjusted p-value <0.00032 and 0.8 power.

#### Analysis of scRNA-seq Data from Primary Screen

##### Processing, Quality Control and Gene Expression Quantification

All 10X scRNA-seq data was processed with the default CellRanger v2.1 pipeline (Zheng et al., 2017) for mapping to the mm10 mouse genome assembly. Gene counts were further analysed with Scanpy (Wolf et al., 2018). To keep only high-quality cells, we filtered out cells with less than 4,000 UMI counts and/or less than 1,600 detected genes, cells with more than 20,000 UMI counts and/or more than 5,000 genes, and cells with more than 5% of UMI reads coming from mitochondrial genes (Figures S2B–S2D). After filtering, 109,061 cells were retained from replicate 1, 118,646 cells from replicate 2 and 107,591 cells from replicate 3. Next, we assigned a sgRNA to each cell using the amplicon sgRNA libraries (see Assignment of sgRNAs to cells below) and we discarded cells that were not uniquely assigned to one sgRNA, resulting in 71,047 cells in replicate 1, 71,188 cells in replicate 2 and 61,729 cells in replicate 3, which corresponds to 203,894 cells in total across all replicate sets (Figure 1C). A gene was considered for downstream analysis if it was detected (UMI count > 0) in at least 10 out of the 203,894 cells that passed filtering. The final dataset after quality control consisted of 20,690 genes. The number of UMIs for each cell and gene were adjusted by the library size in each cell, dividing by the total number of UMIs per cell. Gene expression levels were obtained as log-transformed adjusted UMI counts, scaled by a factor of 10,000. For PCA analysis (Figures 1D, 1E, and S2H), 965 highly variable genes were selected, as implemented in Scanpy (Wolf et al., 2018) with minimum mean of 0.01, maximum mean of 5 and minimum dispersion of 0.5.

##### Assignment of sgRNAs to Cells

Using the amplicon sgRNA libraries, the potential sgRNA sequence (nucleotides 24-43 of the read) was compared to the collection of designed sgRNAs. By taking only exact matches to the white list of sgRNAs, the majority of 475 sgRNA sequences were recovered (470-474, variable from one library to another), and 16% of reads on average (15.3%-16.8% for different libraries) were left unassigned to a sgRNA. To correct for sequencing errors, we allowed a minimum edit distance (Levenshtein distance – 4 edits) between any two sequences of the designed sgRNAs as well as the CROP-sgRNA-MS2 vector sequence surrounding the potential sgRNA in the read. For the reads left unassigned to a sgRNA at this stage, if there was a sgRNA sequence within Levenshtein distance of 1 or 2 and if the upstream 23 nucleotides and downstream 23 nucleotides matched the CROP-sgRNA-MS2 vector sequence with up to 4 edits each, the respective sgRNA was assigned to the read. After this correction procedure, approximately 2% of reads were left unassigned. Cell barcodes detected in the amplicon libraries were then matched with barcodes detected in the regular 10X scRNA-seq libraries. Out of the 317,847 cells that passed quality control across the three transduction replicates in the regular 10X libraries, 249,767 cell barcodes were captured in the amplicon sgRNA libraries (85,993 in replicate 1, 86,671 in replicate 2 and 77,103 in replicate 3). A sgRNA was assigned to a cell if more than 90% of all the amplicon reads containing the sgRNA had the same cell barcode, with standard error of binomial proportion of less than 10% (e.g. more than 8 reads if all the barcodes are associated with the same sgRNA, 13 reads of the same sgRNA if there were more than one sgRNA for a cell barcode, etc). The following table illustrates cell numbers and percentages for each assignment in each replicate (see also Figure 1C):

**Table:** 

Replicate	Total number of cells	Assignment	Number of cells	Percentage
1	85,933	No sgRNA	397	0.46
	85,933	Unique sgRNA	71,047	82.62
	85,933	Two sgRNAs	3,028	3.52
	85,933	Multiple sgRNAs	11,521	13.40
2	86,671	No sgRNA	400	0.46
	86,671	Unique sgRNA	71,118	82.14
	86,671	Two sgRNAs	3,210	3.70
	86,671	Multiple sgRNAs	11,873	13.70
3	77,103	No sgRNA	381	0.49
	77,103	Unique sgRNA	61,729	80.06
	77,103	Two sgRNAs	3,084	4.00
	77,103	Multiple sgRNAs	11,909	15.45

##### Repeat Element Quantification

All occurrences in the genome of repeat sequences from 12 repeat families (LINE-1, LINE-2, ERV1, ERVK, MERVL, Major satellites, Minor satellites, Ribosomal RNA, SINE Alu B1, SINE B2, SINE B4, Telomeric repeats), with each respective genomic locations were downloaded from the UCSC table browser (RepeatMasker, mm10, Nov 2018), concatenated and treated as a reference genome to map the reads discarded by CellRanger pipeline, due to mapping to multiple regions, using SAMtools (Li et al., 2009) and BWA (version 0.7.17-r1188, default parameters) (Li and Durbin, 2009). The following number of reads were discarded by the CellRanger pipeline in each transduction replicate: 253,330,874 reads in replicate 1, 276,401,843 reads in replicate 2, 242,863,617 reads in replicate 3, out of which 38,792,331 (15.31%) in replicate 1, 37,285,351 (13.49%) in replicate 2 and 25,837,444 (10.64%) mapped to repeat elements. LINE-2 elements and Minor satellite repeats were discarded from downstream analysis due to inefficient mapping (see table below). Reads sharing a UMI and a cell barcode were then collapsed in order to get an estimate of the number of molecules for each repeat family in every cell (Figures S3A and S3B).

**Table:** 

Replicate	Repeat family	Number of mapped reads
1	LINE-1	15,930,866
	LINE-2	56
	ERV1	1,049,021
	ERVK	14,783,904
	MERVL	1,275,515
	Major Satellites	305,524
	Minor Satellites	3
	Ribosomal RNA (rRNA)	773
	SINE Alu B1	398,331
	SINE B2	4,583,807
	SINE B4	464,024
	Telomeric repeats	447
2	LINE-1	14,790,530
	LINE-2	86
	ERV1	1,159,969
	ERVK	13,299,950
	MERVL	1,382,105
	Major Satellites	318,816
	Minor Satellites	2
	Ribosomal RNA (rRNA)	846
	SINE Alu B1	457,910
	SINE B2	5,341,151
	SINE B4	533,479
	Telomeric repeats	507
3	LINE-1	9,769,600
	LINE-2	43
	ERV1	952,484
	ERVK	8,865,749
	MERVL	1,049,799
	Major Satellites	173,969
	Minor Satellites	4
	Ribosomal RNA (rRNA)	551
	SINE Alu B1	370,782
	SINE B2	4,226,418
	SINE B4	427,651
	Telomeric repeats	394

##### Multi-omics Factor Analysis + (MOFA+) Model on CRISPRa scRNA-seq Dataset

MOFA (Argelaguet et al., 2018), a hierarchical Bayesian model as implemented in an extension of MOFA (MOFA+) (Argelaguet et al., 2020), was trained on two views: first, the set of 965 highly variable coding genes, and second, the expression levels of eight repeat families (LINE-1, ERV1, ERVK, MERVL, Major satellites, SINE Alu B1, SINE B2 and SINE B4). The ribosomal RNA (rRNA) and telomeric repeats were excluded from the model due to low detection rate (Figures S3A and S3B). The cell-to-sgRNA assignment to group cells based on sgRNA expression was provided to the model in order to take advantage of group-wise sparsity of the model (Figure 2A). Upon interpreting the first five factors using their top loadings of variance explained (Table S4; Figures 2B, 2D, S3C, and S3G), factor 3 was interpreted as a ZGA-like factor based on its top loadings (both coding genes and MERVL repeat) being genes expressed at the time of ZGA (Figures 2B–2D).

##### MOFA+ Model on Deng et al., 2014 (In Vivo Dataset)

MOFA+ (Argelaguet et al., 2020) model was trained on the top 5,000 highly variable coding genes across five developmental stages (zygotes, early 2-cell, mid 2-cell, late 2-cell and 4-cell embryos). Upon interpreting the first three factors using their variance explained across developmental stages, factor 2 was interpreted as a ZGA factor with high values during ZGA stages (mid and late 2-cell embryos) (Figures 2E and S3H) and its top gene loadings corresponding to the top loadings of the CRISPRa ZGA-like factor (factor 3), which are enriched for previously described ZGA genes (Figure S3I; Table S2).

##### Identification of Screen Hits

MOFA+ factor 3 (ZGA-like factor from CRISPRa dataset) values, *Z*, for cells with either of the 228 sgRNAs eliciting any target gene activation (log_2_ fold change of target gene expression to non-targeting sgRNA controls > 0) were compared to MOFA+ factor 3 values for cells with non-targeting sgRNAs, one targeting sgRNA at a time. For each of those sgRNAs, we fitted a linear model $Z_{\left[sgRNA\;A,\;NT\;sgRNAs\right]}\;∼\;I_{\to sgRNA\;A}$ to quantify how much ZGA-like-ness was gained by having a targeting sgRNA in a cell, relative to controls. In this model, a binary indicator for the targeting sgRNA, *I*, was included, being 1 for targeting sgRNAs and 0 for non-targeting sgRNAs. For each sgRNA tested, a fitted effect size (δ) was obtained. P values were obtained from a likelihood ratio test followed by multiple testing adjustment using Benjamini-Hochberg correction. Positive hits were reported at 10% FDR level (Figure 3A; Table S1). These δ values are correlated with higher expression of ZGA-like transcripts (Figures 3B and 3C) and with the fraction of variance explained by MOFA+ factor 3 (Figure S4A).

##### Differential Gene Expression Analysis

For each targeting sgRNA in the screen, respective cells were compared to the set of cells with non-targeting sgRNA controls. A generalised linear model (glm) as implemented in EdgeR was fitted for every gene, and a likelihood ratio test was used to estimate the effect of the targeting sgRNA on the gene’s level of expression (Robinson et al., 2010).

#### Preparation of Bulk RNA-sequencing Libraries

RNA was isolated using RNeasy Mini kit (Qiagen, 74104) and treated with DNaseI (Thermo Fisher Scientific, EN0521) following manufacturer’s instructions.

For SAM and E14 ESCs, opposite strand-specific total RNA libraries (ribozero) were made from 1 μg of DNase-treated RNA using the Sanger Institute Illumina bespoke pipeline and sequenced at 100 bp paired-end on the Illumina HiSeq2500 Rapid Run platform. For CRISPRa (see Table S1 for sgRNAs used) and cDNA validation samples, opposite strand-specific polyA-capture RNA libraries were made from 1 μg of DNase-treated RNA using the Sanger Institute Illumina bespoke pipeline and sequenced at 50 bp single-end on Illumina HiSeq4000.

All bulk RNA-sequencing experiments were performed in three independent replicates, except for SAM ESCs for which two replicates were prepared.

#### Analysis of Bulk RNA-sequencing Data

For processing of all RNA-sequencing data, including those generated in this study but also re-analysis of publicly-available data (Xue et al., 2013; Deng et al., 2014; Barisic et al., 2019), raw FastQ data were trimmed with Trim Galore (www.bioinformatics.babraham.ac.uk/projects/trim_galore/, v0.6.1, default parameters) and mapped to the mouse mm10 genome assembly using Hisat2 (Kim et al., 2019; v2.0.5), as guided by known splice sites taken from Ensembl v96. Hits were filtered to remove mappings with MAPQ scores < 20. Data were quantitated at mRNA level using the RNA-seq quantitation pipeline in SeqMonk software (www.bioinformatics.babraham.ac.uk/projects/seqmonk/) with strand-specific quantification using mRNA probes. For alignments to dCas9-VP64 and MS2-p65-HSF1 exogenous integrations, we constructed an artificial genome and integrated it to mm10 to quantify their expression in relation to the whole transcriptome (Figure S1A).

For alignments to repetitive regions in the genome, we constructed artificial repeat genomes. Repeat annotations were downloaded from the UCSC table browser (RepeatMasker, mm10, Nov 2018). Sequences of the list of repeat element instances were stitched together separated by ‘NNNNN’ to create repeat specific genomes. Trimmed reads from each sample were aligned against all individual repeat genomes using Bowtie2 (Langmead and Salzberg, 2012; v2.3.2). Values reported are cumulative reads mapping to a specific repeat group as percentage of the total read count.

Differentially-expressed genes were determined using EdgeR (FDR < 0.05). Individual CRISPRa of *Patz1, Dppa2*, and *Smarca5* replicate sets were compared to non-targeting sgRNA controls 461 and 462 for differential gene expression (see Table S1 for sgRNA IDs). Differential gene expression for *Dppa2*-GFP^+^, *Smarca5*-GFP^+^ and *Patz1*-GFP^+^ was done against GFP^+^-only controls. The genes that were differentially expressed both by CRISPRa and cDNA overexpression for each target gene were used in Figure 4D. Whole transcriptome correlations (Figure S5D) were calculated using Pearson correlation coefficient on replicate sets.

#### Immunofluorescence and Imaging

Embryos were collected in M2 media (Sigma-Aldrich, MR-015P-5F) containing hyaluronidase (Sigma, H2126), and washed in M2 droplets to remove the cumulus cells. After fixation with 4% PFA (Polysciences, Inc., 18814) for 10 minutes at room temperature, embryos were permeabilised with 0.5% TritonX-100 in PBS for 1 hr and blocked with 1% BSA, 0.05% Tween20 in PBS (BS) for 1 hr. Primary antibodies were diluted in BS and incubated for 1 hr, followed by 1 hr wash in BS. Next, secondary antibodies diluted in BS were incubated for 45 minutes, followed by 30 minutes to 1 hr wash in 0.05% Tween20 in PBS. All incubations were performed at room temperature. Primary antibodies and dilutions used were: rabbit polyclonal anti-PATZ1 (Abcam, Cambridge, UK, ab154025) 1:100, rabbit polyclonal anti-SNF2H (or anti-SMARCA5) (Abcam, ab72499) 1:100 and mouse monoclonal anti-DPPA2 (Merck Millipore, mab4356) 1:200. All secondary antibodies were Alexa Fluor (AF) conjugated and diluted 1:1000: donkey anti-rabbit IgG AF 568 (Invitrogen, A10042) and donkey anti-mouse IgG AF 488 (Invitrogen, A32766). DNA was counterstained with 5 μg/mL DAPI in PBS. Embryos were mounted in fibrin clots. Single optical sections were captured with a Zeiss LSM780 microscope (63× oil-immersion objective) and the images pseudo-coloured using ImageJ2. For visualization, images were corrected for brightness and contrast, within the recommendations for scientific data. Fluorescence co-localization analysis was performed with Volocity 6.3 (Quorum Technologies) in 10 zygotes and 10 two-cell embryos. Pearson correlation coefficient between SMARCA5 and DPPA2 signals were calculated in the area corresponding to the pronuclei or nuclei. The pronuclei in zygotes and nuclei of each blastomere in two-cell embryos were measured separately, with values comparable within the same embryo.

#### Quantitative Reverse Transcription PCR (qRT-PCR)

RNA was isolated using RNeasy Mini kit (Qiagen, 74104) and treated with DNaseI (Thermo Fisher Scientific, EN0521) following manufacturer’s instructions. cDNA was synthesized from 0.5-2 μg of DNAaseI-treated RNA using RevertAid First-Strand cDNA Synthesis Kit (Thermo Fisher Scientific, K1622), and diluted 1:10 prior to qRT-PCR. qRT-PCR was performed in biological triplicate and technical duplicates using Brilliant III SYBR master mix (Agilent Technologies, 600882) and a CFX384 Touch Real-Time PCR Detection System machine (BioRad). Relative levels of transcript expression were quantified by the comparative CT method with normalisation to *Gapdh* levels. Primer sequences are available in Table S6.

### Quantification and Statistical Analysis

Quantification of scRNA-seq data, including quality controls, assignment of sgRNAs to cells and MOFA+ analysis, is described in the section “Analysis of scRNA-seq data from pilot test” and “Analysis of scRNA-seq data from primary screen” under Method Details. The statistical parameters used for power estimations in the primary screen based on the data of the pilot test are described in “Analysis of scRNA-seq data from pilot test screen” under Method Details. Screen hit calling is described in Results and in “Analysis of scRNA-seq data from primary screen” under Method Details; briefly, a regression model based on MOFA+ factor 3 (or ZGA-like factor) values and a binary indicator for the sgRNA targeting activity was fitted for every sgRNA group and p values were obtained from a likelihood ratio test and adjusted using Benjamini-Hochberg correction; positive screen hits were defined at 10% FDR level (Figure 3A; Table S1). Mann-Whitney two-tailed test was applied for comparison of gene and repeat element expression between cells expressing sgRNA hits and cells expressing other sgRNAs; p values are detailed in figure legends (Figures 3B, 3C, S4B, and S4C), with significance established at p value < 0.05. Differential gene expression in the primary screen scRNA-seq data (Figure 3D and S5C; Table S1) was performed with a generalised linear model (glm) as implemented in EdgeR, and a likelihood ratio test was used to estimate the effect of the targeting sgRNA on the gene’s level of expression, as described in “Analysis of scRNA-seq data from primary screen” under Methods Details.

Quantification of bulk RNA-seq data, including those generated in this study but also re-analysis of publicly available data (Xue et al., 2013; Deng et al., 2014; Barisic et al., 2019), is described in “Analysis of bulk RNA-sequencing data” under Method Details. All experiments were performed in triplicate. Differentially-expressed genes to respective controls were determined using EdgeR (FDR < 0.05) and whole transcriptome correlations (Figure S5D) were calculated using Pearson correlation coefficient on replicate sets, as explained in Methods Details. A Mann-Whitney two-tailed test determined significance in repeat element expression between candidate genes tested by arrayed CRISPRa and cDNA overexpression to respective controls (Figures 4D, 5D, and S5E); p values are detailed in figure legends (Figures 4D, 5D, and S5E), with significance established at p value < 0.05.

Quantitative reverse transcription PCR (qRT-PCR) was performed in biological triplicate and technical duplicates. Relative levels of transcript expression were quantified by the comparative CT method with normalisation to *Gapdh* levels. Statistically significant differences to WT GFP^+^ control were evaluated with a homoscedastic two-tailed t-test and p values are reported in figure legends (Figures 6E, 6F, S6A–S6E, and S6G), with significance established at p value < 0.05. The same statistical parameters were applied for quantification of MERVL reporter expression, as analysed by flow cytometry, in *Dppa2* KO cells following *Smarca5* overexpression (Figure S6F).

Fluorescence co-localization analysis in embryos (Figure 6B) is described “Immunofluorescence and Imaging” under Methods Details. A Mann-Whitney two-tailed test was applied on Pearson correlation coefficients and the p value is reported in the figure legend of Figure 6C.

Graphs and illustrations were performed with RStudio, SeqMonk, GraphPad Prism, Microsoft Excel and Illustrator software.

## Supplemental Information

Document S1. Figures S1–S6

Table S1. It Refers to the Design and Analysis of the Pooled CRISPRa Screen with scRNA-Seq Readout, Related to Figures 1, 3, and 5 and STAR Methods This table contains sequence information on the 475 sgRNAs used in the study, target gene, target transcript, and next-generation sequencing results of the pooled sgRNA plasmid library. It also contains information on the CRISPRa scRNA-seq screen dataset, such as number of cells expressing each sgRNA, expression of the target gene, and differential gene expression analyses. All these parameters refer to the three replicates merged. Screen results such as fraction of expression variance explained by MOFA+ factor 3, effect size δ, adjusted p values, hit rank, and whether the sgRNA was considered a hit are also shown in this table. Differential gene expression for each sgRNA as well as information on sgRNAs used for arrayed CRISPRa are also provided here

Table S2. Gene Names of Defined ZGA Signature, Related to Figures 1, 2, and 3 This table contains the gene names of previously identified ZGA genes in Eckersley-Maslin et al., 2016; Hendrickson et al., 2017; Li et al., 2018. The list is a combination of Table S1 from Eckersley-Maslin et al., 2016, Table S8 from Hendrickson et al., 2017, and Table S1 from Li et al., 2018

Table S3. It Refers to the PCA Analysis on the Pooled CRISPRa scRNA-Seq Screen Dataset, Related to Figure 1 This table contains loading values for 965 highly-variable genes in the pooled CRISPRa scRNA-seq screen dataset for the first two PCs (PC1 and PC2) in tab 1, gene ontology enrichment results of the top 50 gene loadings for PC1 in tab 2 and gene ontology enrichment results of the top 50 gene loadings for PC2 in tab 3. Related to Figure 1

Table S4. It Refers to MOFA+ Model Trained on the Pooled CRISPRa scRNA-Seq Screen Dataset, Related to Figure 2 This table contains loading values for 965 highly variable genes in the pooled CRISPRa scRNA-seq screen dataset for MOFA+ factors 1–5

Table S5. It Refers to MOFA+ Model Trained on an *In Vivo* Preimplantation Dataset Across Zygotes, Early Two-Cell, Mid Two-Cell, Late Two-Cell, and Four-Cell Stage Embryos, Related to Figure 2 In the first tab (MOFA+ factor values and *Zscan4c*), the table contains factor values for MOFA+ factors 1–3 and *Zscan4c* normalized expression for each cell analyzed from the Deng et al., 2014 dataset; the second tab (MOFA+ loadings - factors 1–3) contains loading values for the top 5,000 highly variable genes in the Deng et al., 2014 dataset for MOFA+ factors 1–3

Table S6. Oligonucleotide Sequences Used in This Study, Related to STAR Methods

Document S2. Article plus Supplemental Information
