# Supplementary material for: A Single-Cell Transcriptomics CRISPR-Activation Screen Identifies Epigenetic Regulators of the Zygotic Genome Activation Program
Source: Cell Syst. 2020 Jul 22;11(1):25–41.e9. doi: 10.1016/j.cels.2020.06.004 (PMC7383230; doi:10.1016/j.cels.2020.06.004)
Supplement: Document S1. Figures S1–S6 [file mmc1.pdf]

Cell Systems, Volume *11*

Supplemental Information

A Single-Cell Transcriptomics CRISPR-Activation Screen Identifies Epigenetic Regulators of the Zygotic Genome Activation Program

Celia Alda-Catalinas, Danila Bredikhin, Irene Hernando-Herraez, Fátima Santos, Oana Kubinyecz, Mélanie A. Eckersley-Maslin, Oliver Stegle, and Wolf Reik

Figure S1: Related to Figure 1

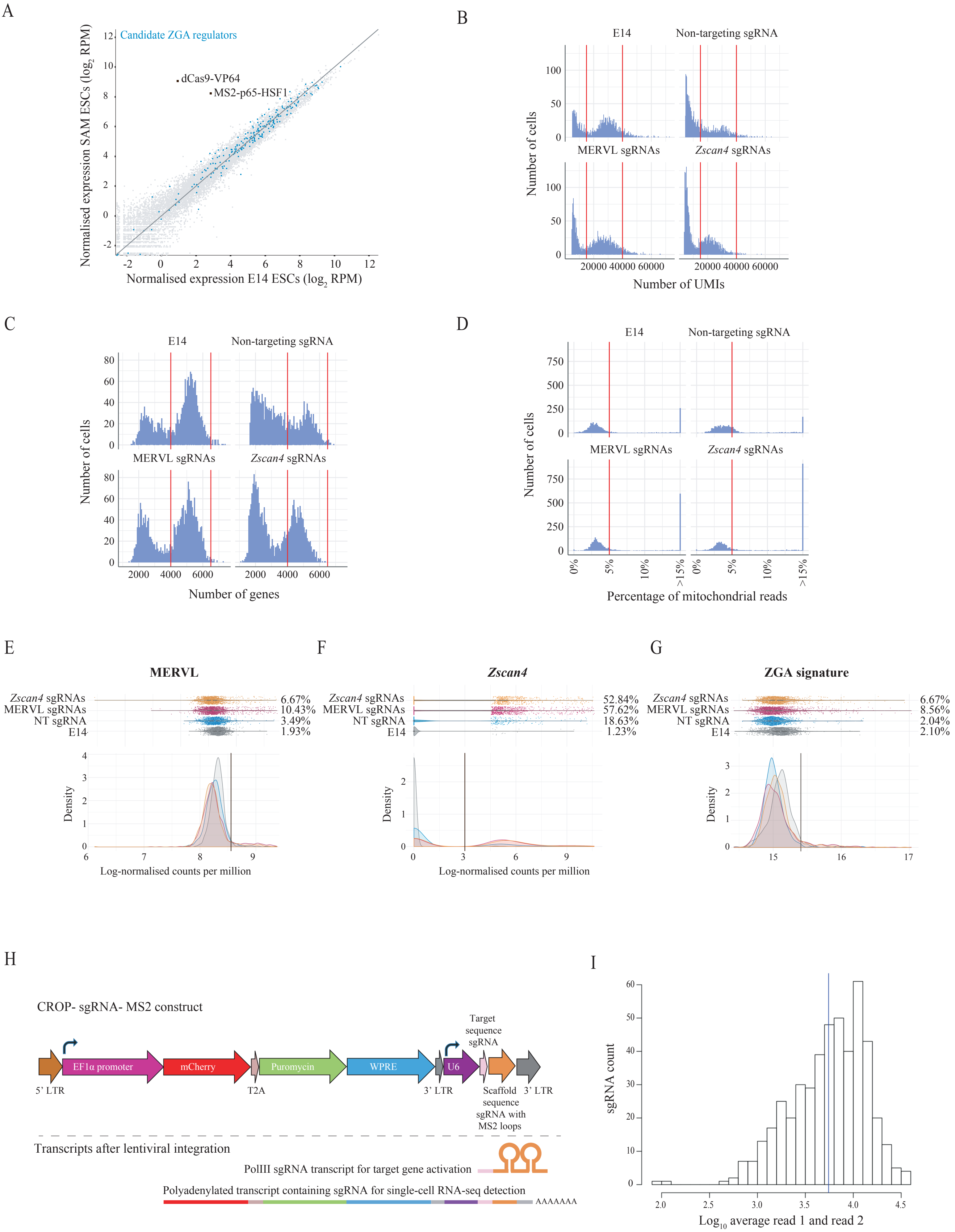

Supplemental Figure 1. Related to Figure 1. A) Scatterplot showing normalised expression levels (log_2 reads per million; RPM) of SAM ESCs against their parental cell line E14, analysed by RNA-sequencing, highlighting dCas9-VP64 and MS2-p65-HSF1 transcripts in black and screening candidates in blue. **B)** Histograms showing number of unique molecular identifiers (UMIs) for each analysed sample; cells within the red bars (15,000<40,000 UMIs) were retained as quality control (QC)-passing cells. **C)** Histograms showing number of detected genes for each analysed sample; cells within the red bars (4,000<6,500 genes) were retained as QC-passing cells. **D)** Histograms showing the percentage of reads from mitochondrial reads for each analysed sample; cells below the red bars (<5%) were retained as QC-passing cells. **E, F)** Dot-plot (upper panels) and density representation (lower panels) of MERVL **(E)** and *Zscan4b/c/d/e/f* (sum of all genes) **(F)** log-normalised expression levels in counts per million in untransduced E14 ESCs (grey), SAM ESCs transduced with a non-targeting sgRNA (blue), SAM ESCs transduced with sgRNAs targeting MERVL LTRs (pink) and SAM ESCs transduced sgRNAs targeting *Zscan4* gene promoters (orange), analysed by 10X Genomics 3' single-cell RNA-sequencing. Reported percentages were calculated with the cells above the vertical black line from the total number of cells in each sample. **G)** Dot-plot (upper panels) and density representation (lower panels) of ZGA genes (as described in Table S2) log-normalised expression in counts per million (sum of all genes) in the samples described in **E)** and **F)**. Reported percentages were calculated with the cells above the vertical black line from the total number of cells in each sample. **H)** Schematic representation of CROP-sgRNA-MS2 lentiviral vector and transcripts produced upon lentiviral reverse transcription and integration in the host genome. **I)** Histogram showing normalised log_{10} average of read 1 and read 2 from next-generation sequencing analysis of the 475 oligo sgRNA library cloned into the lentiviral backbone described in **H)**; representation of 90% of sgRNAs in the library is within 19.9 folds.

Figure S2: Related to Figure 1

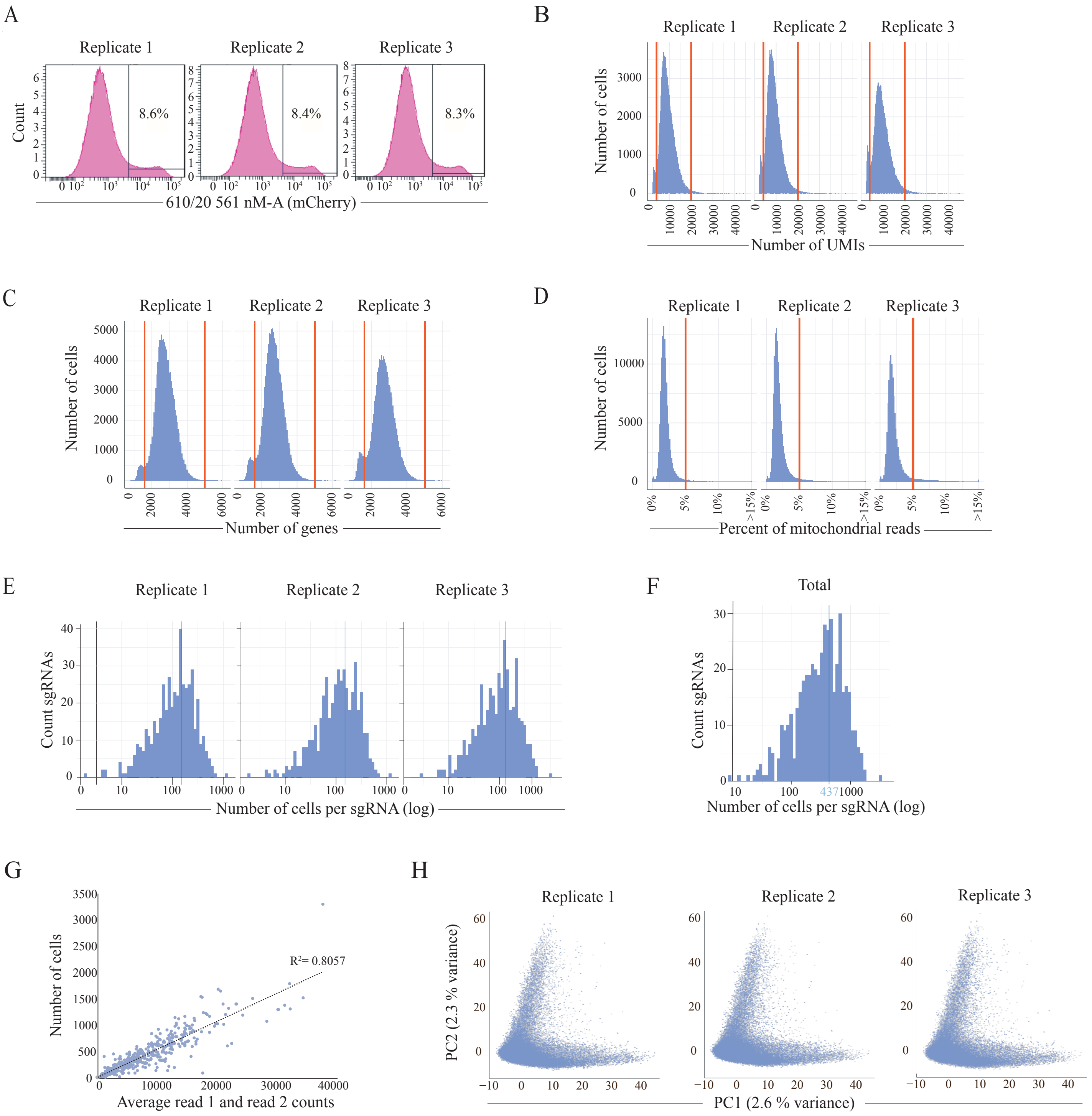

Supplemental Figure 2. Related to Figure 1. A) Histograms showing expression of mCherry analysed by flow cytometry in three replicates of SAM ESCs transduced with the 475 sgRNA lentiviral library at low multiplicity-of-infection (MOI); percentages of cells transduced (expressing mCherry) are shown. **B)** Histograms showing number of unique molecular identifiers (UMIs) for each transduction replicate; cells within the red bars (4,000<20,000 UMIs) were retained as quality control (QC)-passing cells. **C)** Histograms showing number of detected genes for each transduction replicate; cells within the red bars (1,600<5,000 genes) were retained as QC-passing cells. **D)** Histograms showing the percentage of reads from mitochondrial reads for each transduction replicate; cells below the red bars (<5%) were retained as QC-passing cells. **E, F)** Histogram of the number of cells expressing a given unique sgRNA in the 475 sgRNA library for each transduction replicate **(E)** and in the combined dataset across all three replicates **(F)**. The number of cells per sgRNA is depicted in logarithmic scale. The vertical blue line in **F)** represents the average of 437 cells per sgRNA in the dataset. **G)** Scatterplot between the number of cells expressing a given sgRNA in the scRNA-seq activation screen data (y axis) versus the representation of the sgRNA in the cloned oligo library before transduction (average of read 1 and read 2 counts in the NGS libraries- x axis). The sgRNA representation and the number of cells expressing a given sgRNA are correlated ($R^2 = 0.8057$). **H)** Reproducibility across the three transduction replicates: principal component analysis highlighting in each panel the cells from each transduction replicate that contribute to the analysis.

Figure S3: Related to Figure 2

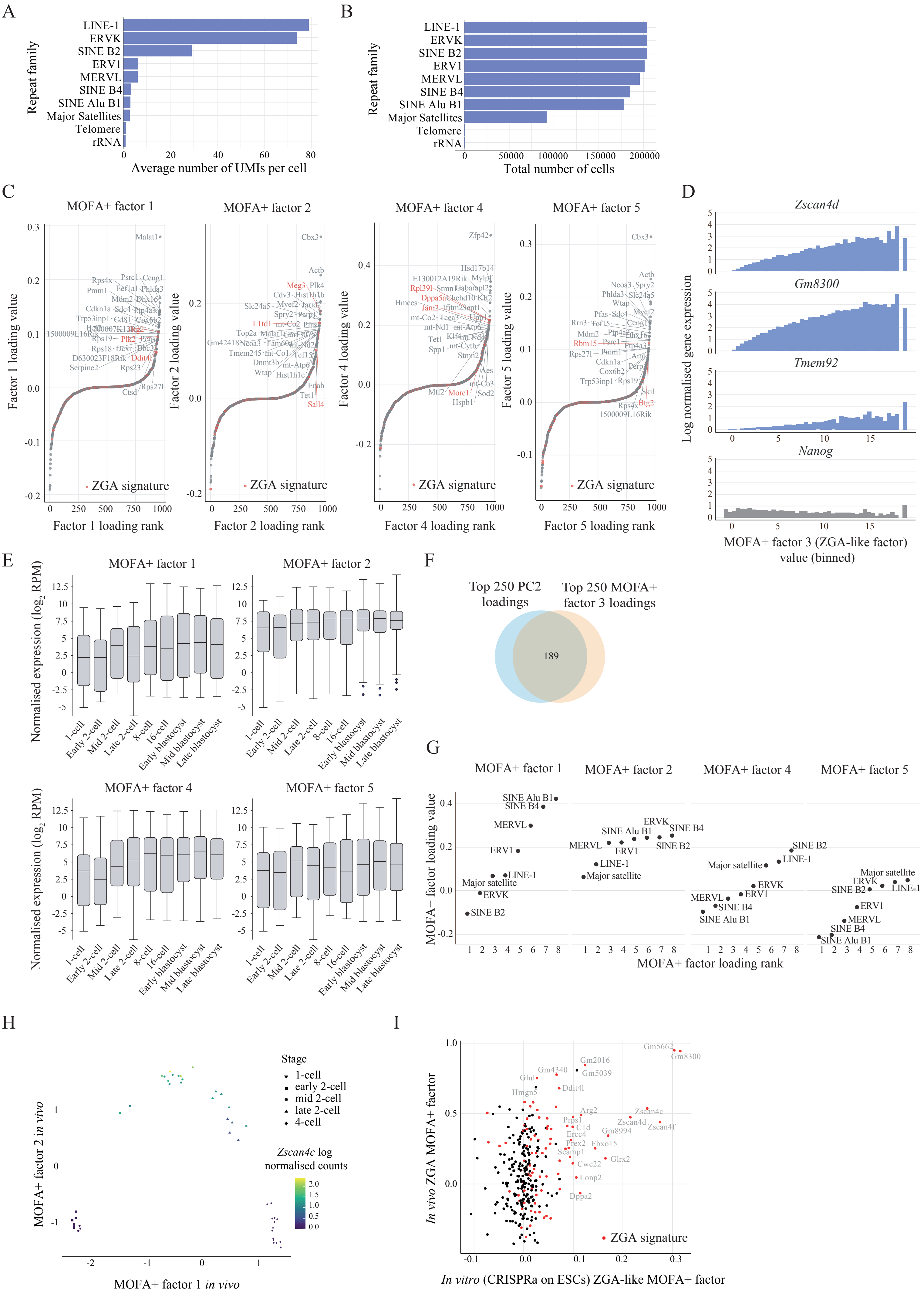

Supplemental Figure 3. Related to Figure 2. A) Average number of UMIs per cell in the combined dataset after quality control for each of the ten repeat families analysed. **B)** Total number of cells in the combined dataset after quality control for which each repeat family is detected (UMI>0), out of a total of 203,894 cells analysed. **C)** Coding genes ranked by their loadings of MOFA+ factors 1, 2, 4 and 5, highlighting in red previously know ZGA genes (as described in Table S2, see also Table S4 for gene loading values). **D)** Normalised expression levels in logarithmic scale for the ZGA markers *Zscan4d*, *Gm8300* and *Tmem92* (blue) and the unrelated gene *Nanog* (grey), for different quantiles of MOFA+ factor 3 (ZGA-like factor) values, showing ZGA markers contribute to the variance explained in this MOFA+ factor. **E)** Box-whisker plots showing normalised expression levels ($\log_2$ reads per million; RPM) for the top 50 gene loadings of MOFA+ factors 1, 2, 4 and 5 during preimplantation development (data analysed from Deng et al., 2014) (see Table S4 for gene loadings). **F)** Venn diagram showing overlap between the top 250 loadings of component 2 identified using conventional principal component analysis (PC2) and the top 250 loadings of MOFA+ factor 3 (ZGA-like factor) (see Table S4 for gene loadings). **G)** Repeat element families ranked by their loadings of MOFA+ factors 1, 2, 4 and 5. **H)** Scatterplot between factor values for MOFA+ factor 1 and factor 2 trained on scRNA-seq data for zygotes, early 2-cell, mid 2-cell, late 2-cell and four-cell stage embryos (Deng et al., 2014) with cells coloured by *Zscan4c* expression. **I)** Scatterplot between gene loadings for factor 2 (or ZGA factor) from MOFA+ model trained on Deng et al., 2014 dataset and gene loadings for factor 3 (or ZGA-like factor) from MOFA+ model trained on our scRNA-seq dataset on CRISPRa-perturbed ESCs, highlighting the top gene loadings for both factors, with those previously know ZGA genes (as described in Table S2) coloured in red (see also Table S5 for gene loading values).

Figure S4: Related to Figure 3

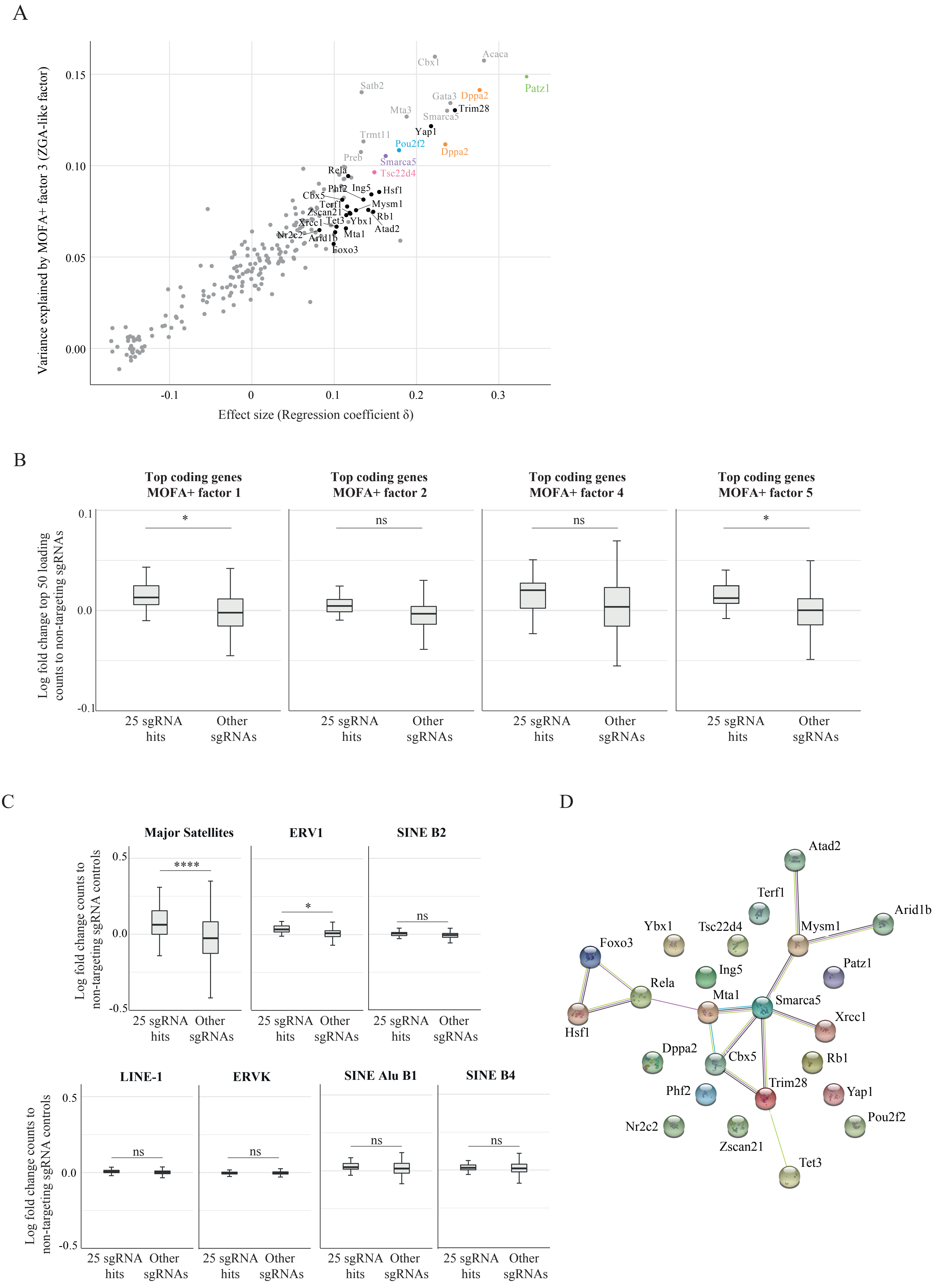

Supplemental Figure 4. Related to Figure 3. A) Scatterplot between the fraction of expression variance (average of coding genes and repeat elements) explained by MOFA+ factor 3 (ZGA-like factor) for each targeting sgRNA (y axis) and effect size (regression coefficient δ). The names of the target genes for sgRNAs identified as hits (see Table S1) are shown, with *Patz1* (green), *Dppa2* (orange), *Smarca5* (purple), *Pou2f2* (blue) and *Tsc22d4* (pink) sgRNA hits highlighted. **B)** Box-whisker plots showing log fold change expression for the top 50 genes associated with MOFA+ factors 1, 2, 4 and 5 (ranked absolute loadings) in cells expressing the 25 sgRNA hits and cells expressing other targeting sgRNAs, compared to cells expressing non-targeting sgRNA controls. Expression is quantified in normalised counts. (*: p-value < 0.05, ns (non-significant): p-value > 0.05; Mann-Whitney two-tailed t-test). **C)** Box-whisker plots showing log fold change of normalised counts of different repeat families in cells expressing the 25 sgRNA hits and cells expressing other targeting sgRNAs, compared to cells expressing non-targeting sgRNA controls (****: p-value < 0.0001, *: p-value < 0.05, ns (non-significant): p-value > 0.05; Mann-Whitney two-tailed t-test). **D)** Network analysis for the 24 screen hits using STRING (https://string-db.org). Nodes represent proteins and edges represent protein-protein associations from known interactions (curated databases – blue, or experimentally determined – pink) or predicted interactions (gene neighbourhood – green or co-expression – black).

Figure S5: Related to Figure 4 and 5

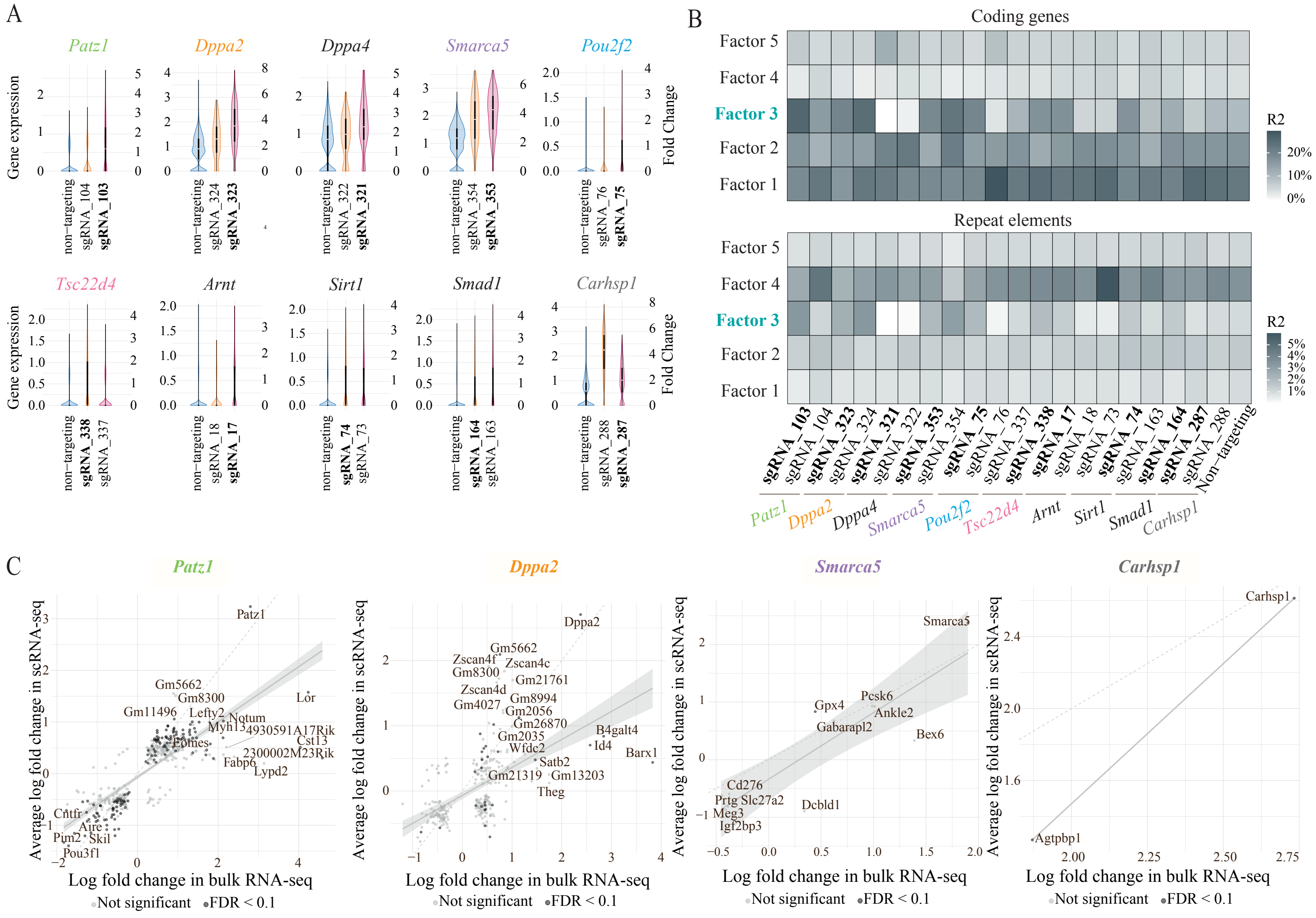

D

		CRISPRa				cDNA			
		Dppa2	*Smarca5*	*Patz1*	*Carhsp1*	*Dppa2*	*Smarca5*	*Patz1*	*Carhsp1*
**CRISPRa**	*Dppa2*	1	0.99615335	0.99515086	0.4625367	0.99281627	0.9863467	0.98981076	0.44171056
	Smarca5	0.99615335	1	0.99493474	0.4625575	0.9943796	0.9895659	0.991365	0.4406198
	Patz1	0.99515086	0.99493474	1	0.4630882	0.99211645	0.9860616	0.9913162	0.44227436
	Carhsp1	0.4625367	0.4625575	0.4630882	1	0.46148068	0.44955397	0.4541936	0.947074
**cDNA**	*Dppa2*	0.99281627	0.9943796	0.99211645	0.46148068	1	0.9930483	0.9941342	0.4434421
	Smarca5	0.9863467	0.9895659	0.9860616	0.44955397	0.9930483	1	0.9922844	0.43217552
	Patz1	0.98981076	0.991365	0.9913162	0.4541936	0.9941342	0.9922844	1	0.43673247
	Carhsp1	0.44171056	0.4406198	0.44227436	0.947074	0.4434421	0.43217552	0.43673247	1

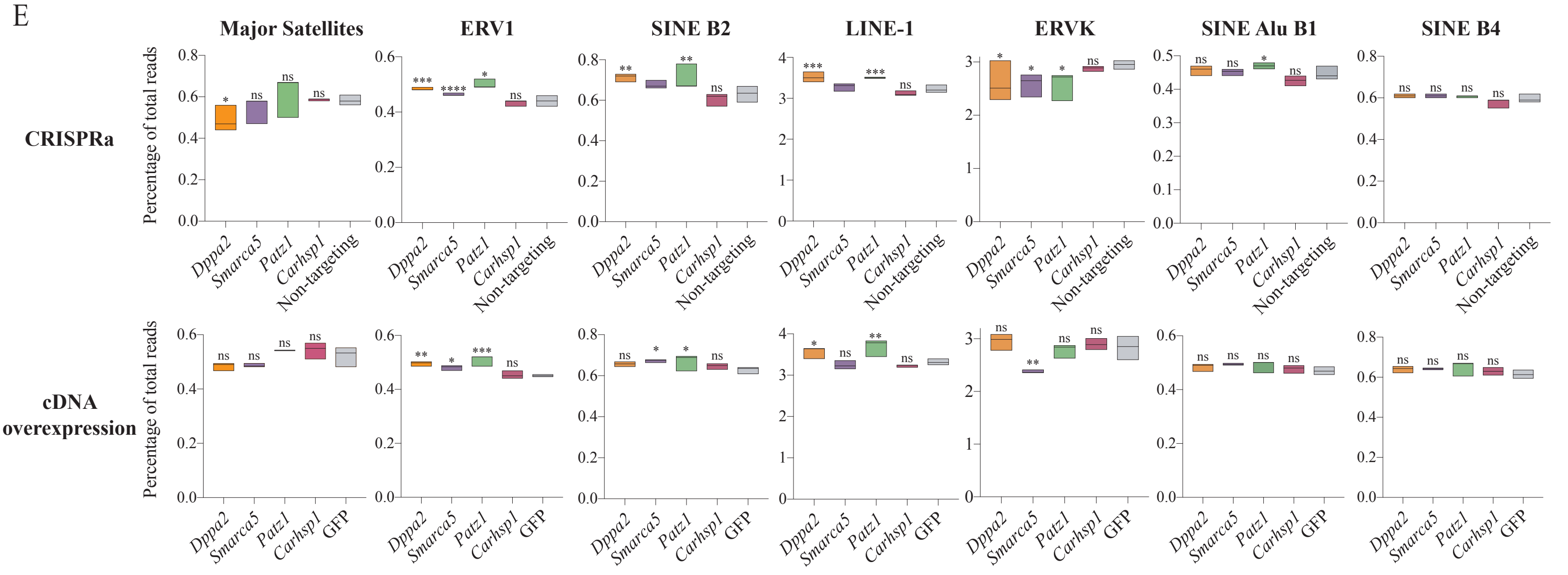

Supplemental Figure 5. Related to Figures 4 and 5. A) Violin plots for *Patz1* (green), *Dppa2* (orange), *Dppa4* (black), *Smarca5* (purple), *Pou2f2* (blue), *Tsc22d4* (pink), *Arnt*, (black), *Sirt1* (black), *Smad1* (black) and *Carhsp1* (grey) showing gene expression (left y axis) and fold change to controls (right y axis) in cells from the screen scRNA-seq dataset expressing their respective targeting sgRNAs (red and orange, see Table S1 for sgRNA identifiers) and in cells expressing non-targeting sgRNA controls (blue). The sgRNA of each target gene used for validation by bulk RNA-sequencing is highlighted in bold. **B)** Fraction of expression variance explained (R^2) by individual MOFA+ factors 1-5 for each data modality (coding genes – upper panel, and repeat elements – lower panel) in targeting sgRNAs for *Patz1* (green), *Dppa2* (orange), *Dppa4* (black), *Smarca5* (purple), *Pou2f2* (blue), *Tsc22d4* (pink), *Arnt*, (black), *Sirt1* (black), *Smad1* (black) and *Carhsp1* (grey) and non-targeting sgRNA controls (see Table S1 for sgRNA identifiers). The sgRNA of each target gene used for validation bulk RNA-seq experiments is highlighted in bold. **C)** Scatterplot of log fold change values of differentially expressed genes estimated based on bulk CRISPRa RNA-sequencing data (FDR<0.1) (x axis) versus log fold change estimates for the corresponding genes in cells expressing the same sgRNA based on the CRISPRa scRNA-seq data (y axis) for the target genes *Patz1*, *Dppa2*, *Smarca5* and *Carhsp1*. Genes that were also differentially expressed in scRNA-seq data (FDR<0.1) are labelled in dark grey whereas those genes differentially expressed in bulk RNA-sequencing data but not in scRNA-seq are labelled in light grey (not significant). For each target gene, a regression line was fitted to highlight the trend. Dashed lines mark $y = x$ line. The sgRNAs used for CRISPRa in bulk RNA-sequencing experiments are described in Table S1. **D)** Heatmap of Pearson correlation coefficients between bulk gene expression profiles of *Dppa2*, *Smarca5*, *Patz1* and *Carhsp1* CRISPRa and cDNA overexpression. The sgRNAs used for CRISPRa in these experiments are described in Table S1. **E)** Box-whisker plots showing expression of different repeat families in percentage of total reads measured by bulk RNA-sequencing after CRISPRa (top panels) and cDNA overexpression (bottom panels) of *Dppa2* (orange), *Smarca5* (purple), *Patz1* (green) and *Carhsp1* (pink) and in controls (two non-targeting sgRNAs for CRISPRa and GFP^+-only for cDNA overexpression, grey). Statistically significant differences to controls are reported as ****: p-value < 0.0001, ***: p-value < 0.001, **: p-value < 0.01, *: p-value < 0.05, ns (non-significant): p-value > 0.05; Mann-Whitney two-tailed test. The sgRNAs used for CRISPRa in these experiments are described in Table S1.

Figure S6: Related to Figure 6

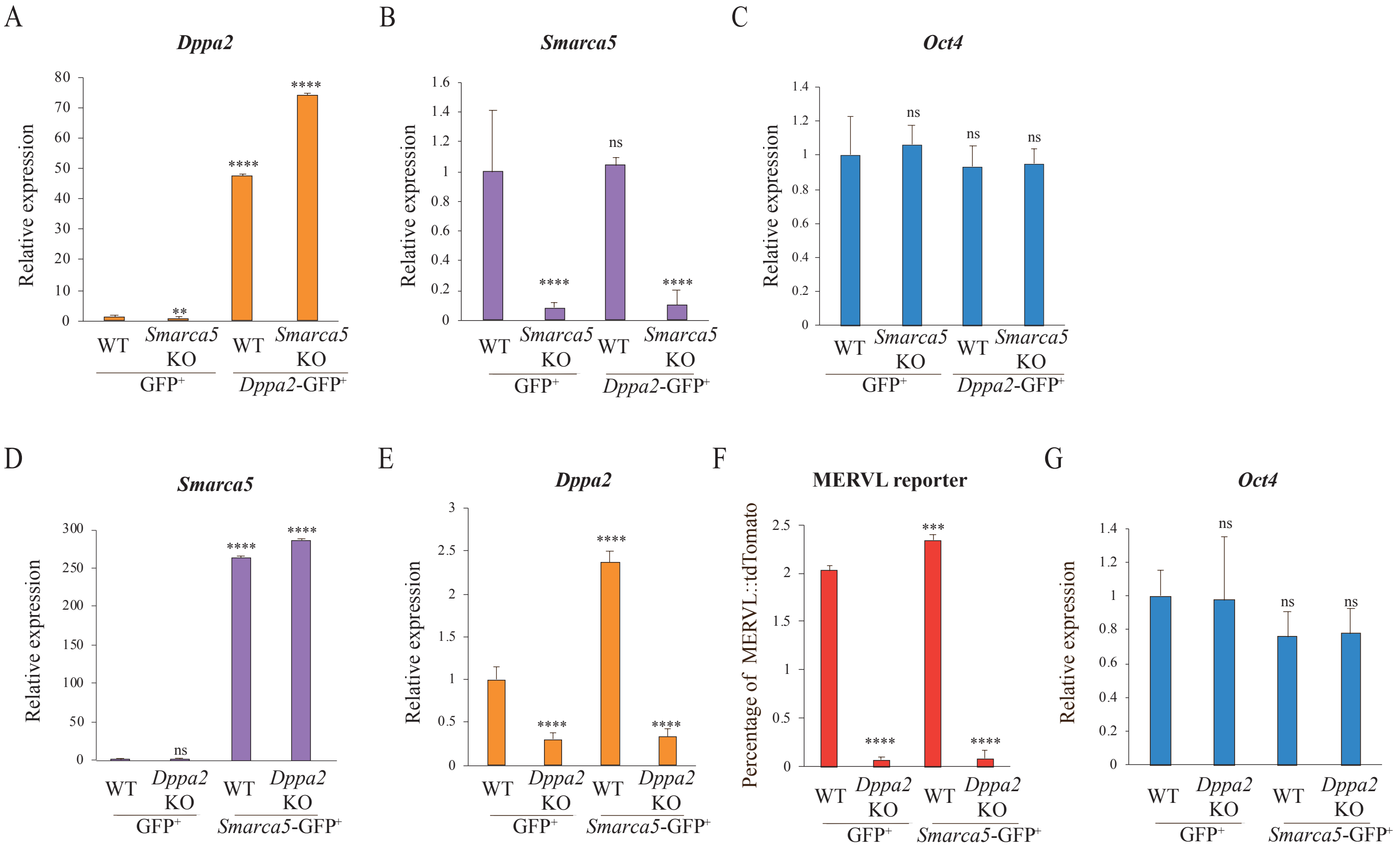

Supplemental Figure 6. Related to Figure 6. A, B, C) Analysis of *Dppa2* (A), *Smarca5* (B) and *Oct4* (C) relative expression levels by quantitative reverse transcription PCR in wild-type (WT) and *Smarca5* knock-out (KO) mouse ESCs after 48 hours transient transfection of GFP or *Dppa2*-GFP and FACS-sorting for GFP^+ cells. **D, E, G)** Analysis of *Smarca5* (D), *Dppa2* (E) and *Oct4* (G) relative expression levels by quantitative reverse transcription PCR in WT and *Dppa2* KO mouse ESCs after 48 hours transient transfection of GFP or *Smarca5*-GFP and FACS-sorting for GFP^+ cells. **F)** Percentage of GFP^+ cells expressing a MERVL::tdTomato reporter in WT and *Dppa2* KO mouse ESCs after 48 hours transient transfection of GFP or *Smarca5*-GFP, analysed by flow cytometry. In all panels, relative expression levels are normalised to WT cells transfected with GFP and sorted for GFP^+; data is shown as mean plus standard deviation of three biological replicates; statistically significant differences to WT GFP^+ control are reported (**: p-value < 0.01, ***: p-value < 0.001, ****: p-value < 0.0001, ns (non-significant): p-value > 0.05; homoscedastic two-tailed t-test).
